# NIRFASTerFF: an accessible, cross-platform Python package for fast photon modeling

**DOI:** 10.1117/1.JBO.30.11.115001

**Published:** 2025-11-03

**Authors:** Jiaming Cao, Samuel Montero-Hernandez, Rickson C. Mesquita, Adam T. Eggebrecht, Hamid Dehghani

**Affiliations:** a University of Birmingham, School of Computer Science, Birmingham, United Kingdom; b Washington University School of Medicine, Mallinckrodt Institute of Radiology, St. Louis, Missouri, United States

**Keywords:** diffuse optics, finite element method, algorithm, toolbox, mathematical modeling, parallel computing

## Abstract

**Significance:**

Accurate and efficient photon modeling plays an essential role in the rapidly developing field of diffuse optical imaging, whereby the use of model-based analysis and image reconstruction can provide both educational and research benefits.

**Aim:**

NIRFASTerFF is a cross-platform (Linux, macOS, and Windows) Python package for finite element method (FEM)-based light propagation modeling, supporting continuous-wave, frequency-domain, and time-resolved data for both exogenous and endogenous optical imaging applications. It also enables modeling of the autocorrelation function ($G_{1}$) for diffuse correlation spectroscopy. Validation is performed through comparison with the original NIRFAST and gold-standard Monte Carlo simulations.

**Approach:**

NIRFASTerFF incorporates highly parallelized FEM solvers for efficient computation on both CPU and GPU, leveraging OpenMP and CUDA acceleration. To support image reconstruction tasks, voxel-based interpolation of the optical fluence is implemented, providing a flexible and accurate representation of the forward solution suitable for inverse problem formulations.

**Results:**

Compared with its predecessor, NIRFASTer, the optimized algorithms provide a performance boost of 25% to 45% on GPU and up to 20% on CPU, and the results show good agreement with both Monte Carlo and analytical solutions.

**Conclusion:**

The NIRFASTerFF package provides a fast and license-free tool for photon modeling and can further streamline Python-based data processing in diffuse optical imaging, benefiting the biophotonics community.

## 1 Introduction

Optical imaging of physiology using functional near-infrared spectroscopy (fNIRS) and diffuse optical tomography (DOT) has become increasingly widely used and has been rapidly maturing in recent decades. The technology has been used to measure a range of tissues, including the brain, breast, and muscle, both in laboratory and clinical settings.^1^^,^^2^

fNIRS uses near-infrared (NIR) light to illuminate the tissue, and the exiting light intensity can be used to infer the optical properties of the tissue, which can subsequently be used to estimate physiological properties (e.g., functional activation within the brain and tissue characterization). When using an array of overlapping light sources and detectors, it is possible to further reconstruct spatially varying optical properties and subsequently physiological properties, tomographically, referred to as DOT.^3^ In DOT, one key step is to model the propagation of light in the medium to allow model-based image reconstruction, often through nonlinear optimization.^4^ Although Monte Carlo simulation remains the “gold standard” and the GPU-accelerated algorithms have significantly reduced computational time,^5^^,^^6^ it can still be undesirably slow when, for example, there is a large number of light sources to simulate for very large models, which is the case for high-density DOT.^7^ This is mainly because of the stochastic nature of Monte Carlo methods, where a large number of photons must be simulated to reduce statistical noise. For the same reason, it is particularly challenging to accurately estimate the photon distribution at low-fluence locations because the photon count is low and the noise is consequently high.

Alternatively, the photon modeling problem can be solved numerically using the Boltzmann equation for photon transport, which can typically be simplified into the standard diffusion equation in applications where the medium is highly scattering, such as tissue optics.^8^ It has been well-established that the finite element method (FEM) can be used to solve the diffusion equation^9^^,^^10^ and that accelerated algorithms can be used to solve the problem very efficiently.^7^ In comparison to Monte Carlo methods, a great advantage of FEM-based solutions is that they do not suffer from statistical uncertainty, which can be beneficial when estimating photon distribution at low-fluence locations.

Toast++^11^ is one of the most commonly used tools for FEM-based photon modeling. With its core algorithms implemented in C++, the software provides interfaces in both MATLAB and Python. However, Toast++ does not support diffuse correlation spectroscopy (DCS) modeling, and its support for fluorescence problems is limited. More importantly, the GPU implementation of the algorithms is not fully optimized, with only limited parallelization and simplistic choices of preconditioners (see Sec. 2.2.1 for details). Near Infrared Fluorescence and Spectral Tomography (NIRFAST)^10^ and its GPU-accelerated successor, NIRFASTer,^7^^,^^12^ are also commonly used tools for FEM-based photon modeling. Implemented in MATLAB and C/C++, the toolboxes provide an easy-to-use pipeline for photon modeling in a range of problems, including DCS. The support for fluorescence problems (e.g., time-resolved fluorescence), however, remains limited, and GPU-accelerated algorithms do not fully utilize the processing power of modern GPUs, with their efficiency still needing improvement.

This paper presents an accessible, cross-platform Python package for efficient FEM-based photon modeling, named NIRFASTerFF (NIRFASTer Fast and Furious). This is a new generation of the widely used, MATLAB-based photon modeling toolboxes NIRFAST and NIRFASTer, with richer functionality and significantly more efficient algorithms, as will be quantified in Sec. 4.

A notable change as compared to the previous generations of NIRFAST(er) is the use of Python as the scripting language. Doing so provides several benefits: (1) Python is nonproprietary and is, therefore, more accessible to a broader audience; (2) with the emergence of Python-based fNIRS/DOT data processing tools, e.g., MNE-NIRS,^13^ NeuroDOTpy,^14^ and Cedalion,^15^ offering a photon modeling tool in the same language can better streamline the user workflow; and (3) it can also better streamline the machine learning studies, which have seen considerable growth in recent years and heavily depend on Python packages, e.g., TensorFlow and PyTorch.^16^^–^^18^

**Fig. 1:**
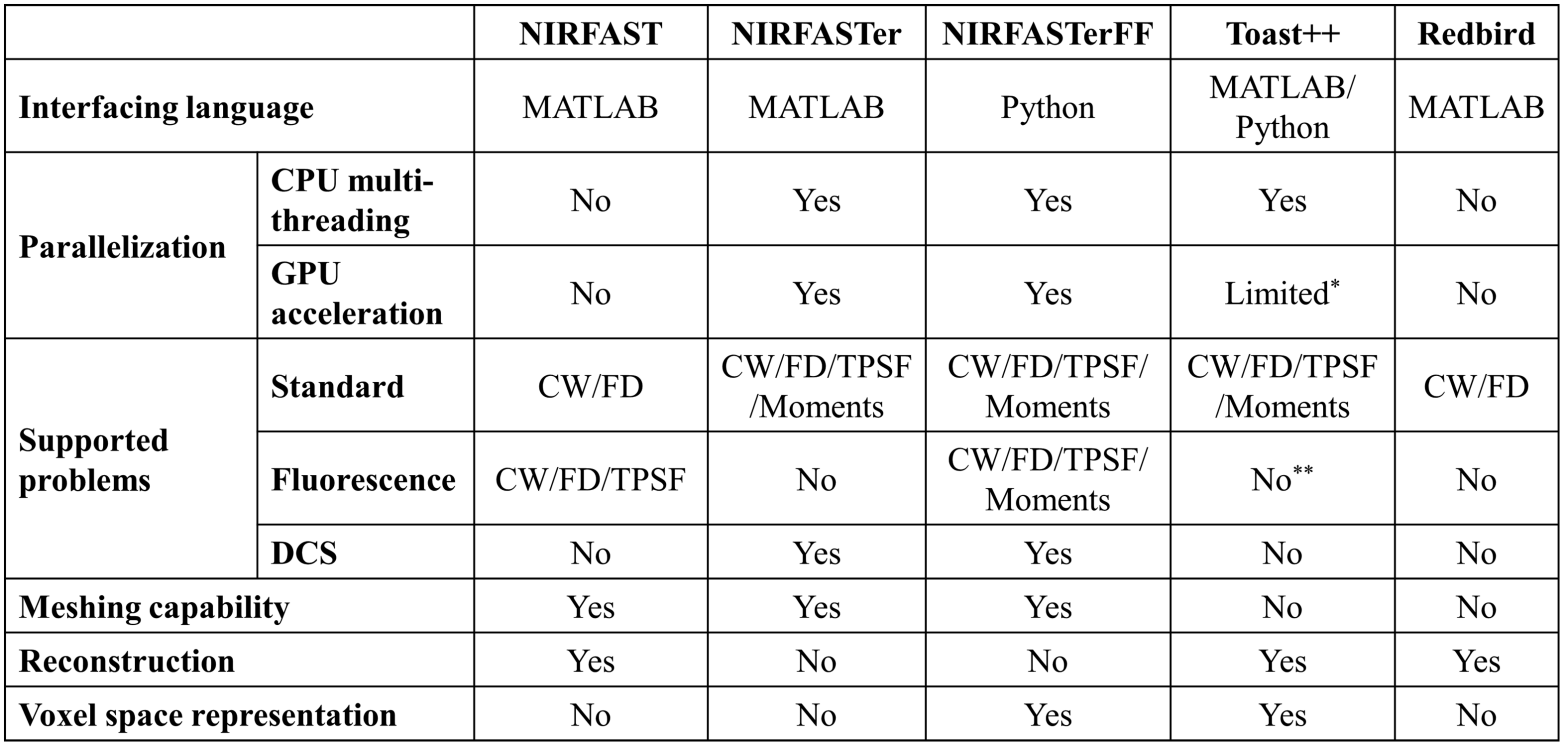
Comparison of key functionalities and implementations between several commonly used FEM-based photon simulators. *High-level CUDA acceleration of some algorithms is implemented but not shipped with the precompiled libraries. **Technically, it is possible to generate fluorescence data as demonstrated in one of the example codes, but no direct support is available.

Functionally, in addition to the classic fNIRS/DOT problem where fluence at a single wavelength is modeled (here referred to as the “standard problem”), NIRFASTerFF has also added full support for the fluorescence problem (fluence, time-resolved, and Jacobian) and DCS. Along with the FEM-based algorithms, analytical solutions to standard and DCS problems in the semi-infinite medium are also included, which, although not novel, add a significant degree of functionality. Although the fluorescence problem was supported in the original NIRFAST and DCS functionality was made available in NIRFASTer, NIRFASTerFF further introduces Mellin transform-based direct moment calculation for time-resolved fluorescence problems (Sec. 2.1.4) and, more importantly, features much faster algorithms. Theoretically, any diffusion equation solver can be extended to support other types of problems (e.g., fluorescence and DCS),^11^ but NIRFASTerFF provides specialized modules and wrapper functions for these problems so that they can be conveniently solved in a “one-liner” fashion. A detailed comparison between some commonly used FEM solvers, including the NIRFAST family,^10^^,^^12^ Toast++,^11^ and Redbird,^19^ is summarized in Fig. 1.

## 2 Methods

**Fig. 2:**
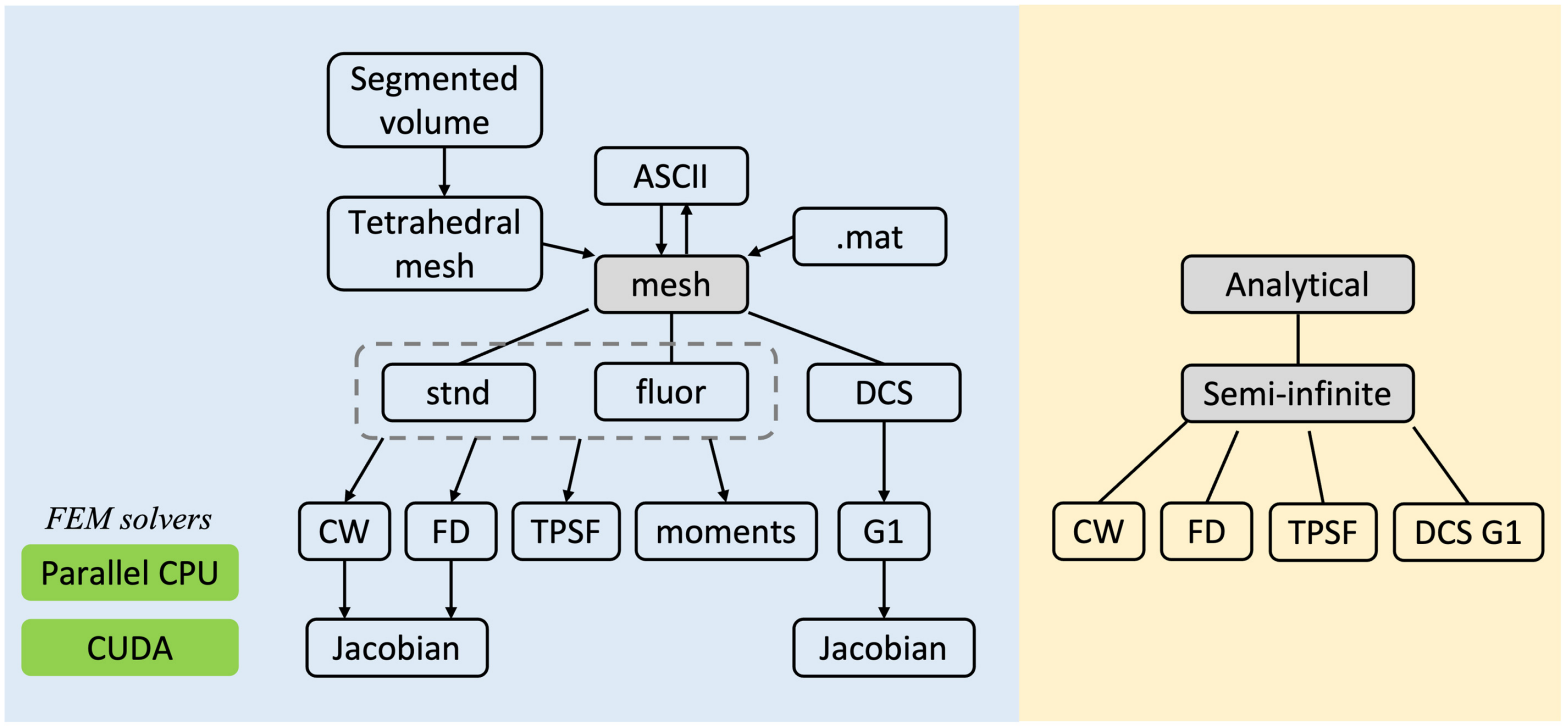
Organization of the NIRFSTerFF package, which provides forward modeling using both FEM (in blue background) and analytical solutions (in yellow background). The FEM part of the package is centered around a mesh object, which can be one of the three types: standard (“stnd”), fluorescence (“fluor”), and DCS. Construction, reading, and saving of the mesh, as well as FEM-based forward modeling and Jacobian calculation, are implemented as methods of the object. CW: continuous-wave, FD: frequency-domain, TPSF: temporal point spread function, and G1: temporal auto-correlation function.

The organization, as well as the supported problems of the package, are illustrated in Fig. 2 and will be discussed in detail in the subsequent sections.

### 2.1 Theory

#### 2.1.1 Standard problem

The standard problem calculates the fluence in a medium at a given excitation wavelength. This is done by solving the standard diffusion equation in the frequency domain^4^^,^^8^

$$-\nabla \cdot (\kappa (r)\nabla \Phi (r,\omega ))+\mu_{a}(r)\Phi (r,\omega )+\frac{i\omega }{c(r)}\Phi (r,\omega )=q(r,\omega ),$$ (1)

where r is the location vector, $\mu_{a}(r)$ is the absorption coefficient, ω is the modulation angular frequency, Φ(r,ω) is the fluence rate, q(r,ω) is the light source, $\kappa (r)=1/3(\mu_{a}(r)+\mu_{s}^{\prime }(r))$, and $\mu_{s}^{\prime }(r)$ is the reduced scattering coefficient. The same equation is used for both frequency-domain (FD) and continuous-wave (CW) problems, where in the latter case, ω=0. The time domain version simply requires replacing iω with the time differential operator, $\frac{\partial }{\partial t}$. The diffusion equation is then solved subject to the Robin-type boundary condition^20^

$$\Phi (m)+2\beta \kappa (m)\frac{\partial \Phi (m)}{\partial \nu }=0,$$ (2)

where m is a point on the boundary, ν is the outer normal at m, and β is a scalar related to the boundary reflection. When the problem is discretized using FEM, it effectively becomes solving the following linear system

(A+iωB)Φ=Q, (3)

where $A\in R^{n\times n}$ and $B\in R^{n\times n}$ (n being the number of nodes in the discretized space) are sparse symmetric positive definite system matrices,^11^ and $\Phi \in R^{n\times s}$ and $Q\in R^{n\times s}$ (s being the number of light sources) are the fluence and source terms, respectively.

#### 2.1.2 Fluorescence problem

The fluorescence problem can be formulated in a similar way, except that it is now a pair of coupled diffusion equations modeling the excitation (denoted by superscript x) and fluorescence re-emission (denoted by superscript m), respectively^21^

$$-\nabla \cdot (\kappa^{x}(r)\nabla \Phi^{x}(r,\omega ))+\mu_{a}^{x}(r)\Phi^{x}(r,\omega )+\frac{i\omega }{c(r)}\Phi^{x}(r,\omega )=q^{x}(r,\omega )\;-\nabla \cdot (\kappa^{m}(r)\nabla \Phi^{m}(r,\omega ))+\mu_{a}^{m}(r)\Phi^{m}(r,\omega )+\frac{i\omega }{c(r)}\Phi^{m}(r,\omega )=\Phi^{x}(r,\omega )\frac{\eta (r)\mu_{af}(r)}{1+i\omega \tau (r)},$$ (4)

where η(r), $\mu_{af}(r)$, and τ(r) are the quantum efficiency, absorption coefficient, and lifetime of the fluorophore, respectively. In practice, it is common to use $\gamma (r)≔\eta (r)\mu_{af}(r)$ as a lumped parameter because it is in general difficult to distinguish between the contributions of the two terms. In this formulation, fluorescence is assumed to be only linearly dependent on the excitation light, and the more complex phenomena, e.g., fluorescence saturation and radiation trapping, are not considered. This approximation holds for weak fluorescent concentrations, which is typically true in biological systems.^22^

#### 2.1.3 DCS problem

In DCS problems, NIRFASTerFF assumes Brownian-type motion, and the forward problem is treated as the correlation diffusion equation in the continuous-wave domain in the following form:^4^

$$-\nabla \cdot (\kappa (r)\nabla G_{1}(r,\tau ))+(\mu_{a}(r)+2\alpha (r)D_{b}(r)\mu_{s^{\prime }}(r)k_{0}^{2}\tau )G_{1}(r,\tau )=q(r),$$ (5)

where $G_{1}$ is the temporal auto-correlation function for the electric field, τ is the delay time at which auto-correlations are calculated, α(r) is the fraction of photon scattering events that occur from moving particles in the medium, $D_{b}(r)$ is the constant related to effective diffusion, and $k_{0}$ is the wavenumber (i.e., $k_{0}=2\pi /\lambda$, where λ is the light wavelength). In practice, it typically suffices to treat $\alpha (r)D_{b}(r)≔\alpha D_{b}(r)$ as a lumped parameter because α(r) is typically difficult to quantify. When $G_{1}$ is normalized by the CW field (i.e., solution at τ=0), the quantity is referred to as the normalized electric field auto-correlation function, denoted as $g_{1}$.

As the three problems (including their time domain counterparts^23^) have very similar mathematical expressions, they can, therefore, all be reduced to the same simple linear form as in Eq. (3). The key to solving these forward problems efficiently is, consequently, the implementation of high-performance solvers for large linear systems; the design of which will be discussed in detail in Sec. 2.2.1, and their performance benchmarked in Sec. 4.

#### 2.1.4 Time-resolved data

For both standard and fluorescence models, NIRFASTerFF provides two different ways of modeling time-resolved (TR) data: calculating the temporal point spread function (TPSF), denoted as f(t), and directly calculating the normalized moments. The zeroth moment is defined as

$$\langle t^{0}\rangle =\int_{0}^{\infty }f(t)dt,$$ (6)

and the n’th normalized moment is defined as

$$\langle t^{n}\rangle =\frac{\int_{0}^{\infty }t^{n}f(t)dt}{\left\langle t^{0}\right\rangle }\;n=1,2,\ldots .$$ (7)

Note that the definitions leverage the fact that f(t)=0 for t<0. Physically, $\left\langle t^{0}\right\rangle$ is the intensity (equivalent to fluence in the standard problem), $\mu =\langle t^{1}\rangle$ is the mean time of flight, and $V=\langle t^{2}\rangle -(\langle t^{1}\rangle {)}^{2}$ is the variance of the TPSF. For standard models, the TPSF calculation and the Mellin transform-based direct moment calculation follow the methods proposed by Arridge and Schweiger.^9^^,^^23^ When the exact distribution of the TPSFs is not of concern, it is usually more desirable to directly calculate the normalized moments. This is because, in the direct moment calculation, the linear system is solved only once for every moment, whereas the TPSF calculation requires solving the system for every time point. In a typical application, at least hundreds of time points are needed for an accurate simulation of the TPSF,^9^ which is computationally very expensive.

The calculation of TPSF (both excitation and fluorescence re-emission) in the fluorescence model follows the method proposed by Zhu et al.^24^ Direct moment calculation in the fluorescence model using the Mellin transform is also implemented for both excitation and fluorescence re-emission, which is analogous to the standard problem^23^ and is detailed in Appendix A. Similar to the standard mesh, it is more desirable to use the direct moment calculation if the exact distribution of the TPSFs is not of concern as it is computationally much less expensive.

### 2.2 Implementation

#### 2.2.1 FEM solvers

As discussed in Sec. 2.1, the key to all the FEM forward modeling problems is implementing appropriate solvers for the linear system in Eq. (3). Directly solving the linear system is, however, often computationally intractable in large systems (e.g., the human brain model, where it is common to have ∼500,000 nodes) due to the high memory cost.^7^

This can be mitigated by using iterative algorithms. In NIRFASTerFF, two types of parallelized (using OpenMP and CUDA) iterative solvers are implemented on both CPU and GPU: the preconditioned conjugate gradient (PCG) algorithm and the preconditioned biconjugate gradient stabilized (BiCGStab) algorithm. In the CPU version, the solvers are invoked for every light source in a loop. In the GPU version, the algorithms are further accelerated by solving for multiple light sources simultaneously in batches.

**Fig. 3:**
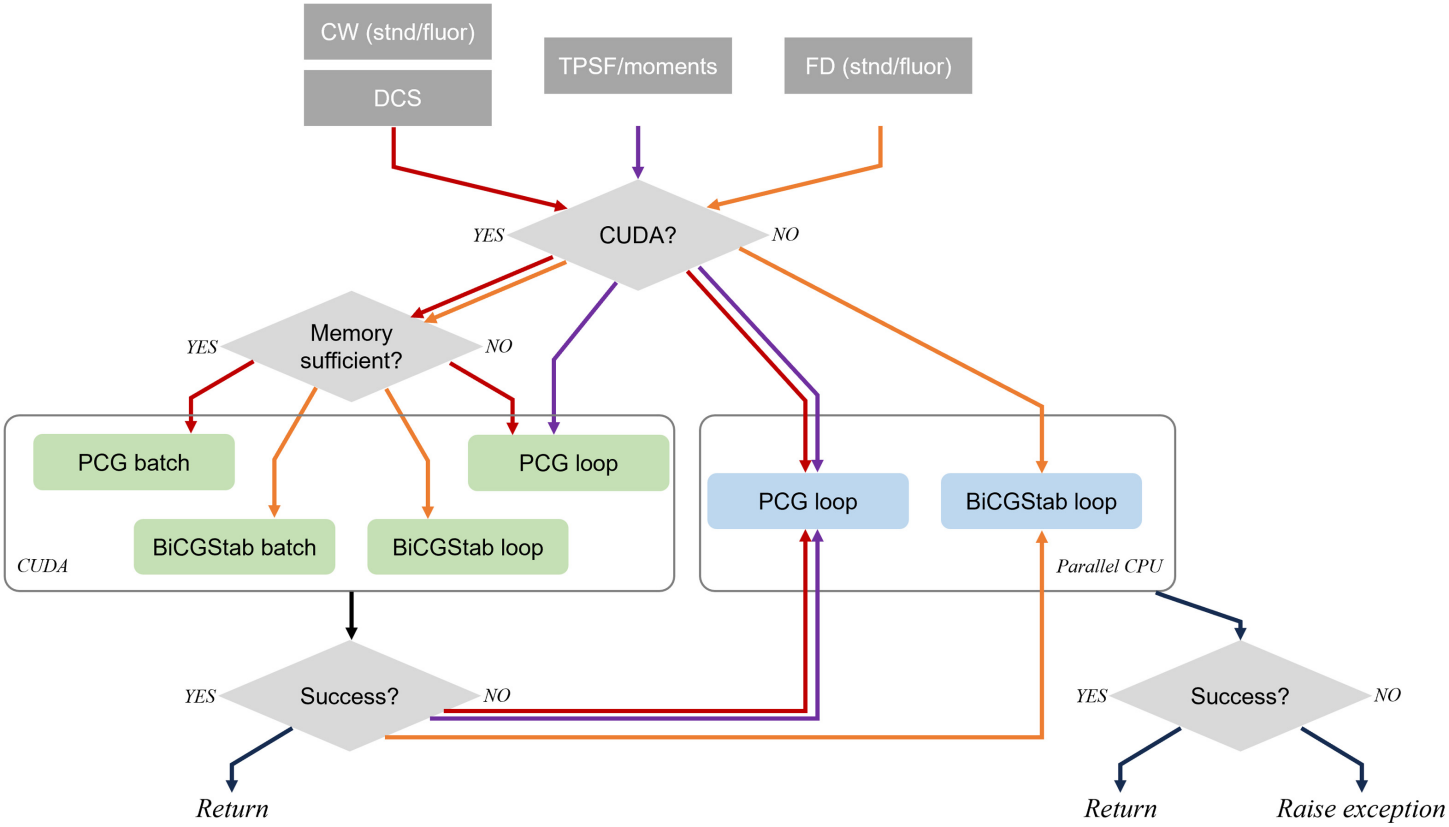
Automatic selection of algorithms depending on the problem and the hardware capabilities. Different problem types are indicated by arrows in different colors. The BiCGStab solvers are used only for FD problems (both standard and fluorescence), and the PCG algorithms are chosen otherwise. Unless explicitly specified, the package always prioritizes the appropriate (i.e., BiCGStab or PCG, depending on the problem) CUDA solvers, among which the batched versions will be first attempted. If CUDA is available, but the GPU does not have sufficient memory, the looped versions will be used instead. If the CUDA solvers fail, or CUDA is unavailable, or the use of CPU is explicitly specified, the CPU implementation of the appropriate solvers (for the problems) will be used. If all attempted solvers fail, an exception will be raised.

This contrasts with the implementations in the previous generations of NIRFAST(er), where the same looped BiCGStab algorithm was used for all problems, and leads to a significant speed boost (see the benchmark results in Sec. 4). The appropriate algorithm is automatically chosen depending on the problem and the hardware, as illustrated in Fig. 3.

##### Primer on preconditioners

Consider a large, sparse, square linear system to be solved using Krylov subspace methods (e.g., BiCGStab and conjugate gradient), Ax=b, it is usually beneficial to multiply both sides by $M^{-1}$ and transform the problem into

$$M^{-1}\mathrm{Ax}=M^{-1}b,$$ (8)

if $M^{-1}A$ is better conditioned than A, and matrix M is referred to as the preconditioner.^25^ It can be seen intuitively that if M is chosen such that $M^{-1}\approx A^{-1}$, the convergence can be accelerated by not only reducing the condition number of $M^{-1}A$ but also providing the algorithm with a good initial guess. One commonly used family of preconditioners is incomplete factorizations, most notably incomplete Cholesky and incomplete LU, where M=LU≈A, with L and U being lower- and upper-triangular matrices that resemble the corresponding full factorizations (e.g., Cholesky or LU) but sparser.^11^ In every iteration of BiCGStab and PCG, it is necessary to solve a (or two, if BiCGStab) linear system with M on the left-hand side, Mz=r, where z and r are some intermediate vectors resulting from the algorithms.^26^^,^^27^ Leveraging the sparse decomposition, the system(s) can be solved efficiently by pivoting the two triangular matrices.

In NIRFASTerFF, the preconditioner is chosen from a different family, where a sparse representation of $M^{-1}$ is explicitly calculated, such that solving the intermediate linear system(s) simplifies into a sparse matrix-vector multiplication, which is even faster than pivoting the triangular matrices. This simplification can lead to considerable accelerations when, e.g., the system is large, or a large number of iterations are needed. The particular one chosen is called the factorized sparse approximate inverse (FSAI), which, following algorithm 5 in Ferronato’s review work,^25^ targets to find a sparse matrix G, such that $M^{-1}=G^{T}G\approx A^{-1}$. It is crucial to choose an appropriate nonzero pattern $S_{L}$ (i.e., the subset of elements in A used to construct the G matrix) for good performance, and the choices will be discussed in the following two paragraphs.

##### BiCGStab algorithm

The implementation of the BiCGStab algorithm follows the same idea as NIRFASTer, where when constructing the FSAI preconditioner, the nonzero pattern $S_{L}$ was chosen to be, for each row i, the locations of the three largest values among $\left[A_{i,1},\ldots ,A_{i,i}\right]$.^7^ This was empirically determined to have a good balance between the number of iterations before convergence and the computational cost per iteration (i.e., sparsity of $M^{-1}$).

##### PCG algorithm

One may notice that when ω=0 in Eq. (3) (e.g., when calculating CW data), the linear system becomes real and positive definite. This allows the use of the more efficient PCG solver,^26^ which, in comparison to BiCGStab,^27^ requires two fewer matrix-vector multiplication steps, and is therefore less expensive per iteration. However, it was also observed that PCG took more iterations to converge when the same preconditioner as in BiCGStab was used. To accelerate the algorithm, the following preconditioners were chosen for the PCG algorithms: $S_{L}$ for the CPU version was chosen to be, for each row i, the diagonal AND the locations of the four largest values among $\left[A_{i,1},\ldots ,A_{i,i-1}\right]$. For the CUDA version, $S_{L}$ was chosen to be the entire lower triangular (including the diagonal) part of A.

The choices made in the NIRFASTerFF were made empirically and heuristically, showing good performance on several computers. In general, however, the optimality of the preconditioner is difficult to guarantee as it depends heavily on the hardware and the problem itself. In the case of photon modeling, this can be dependent on, e.g., the size of the mesh and the optical properties. A denser preconditioner is used in the CUDA version because the extra cost per iteration is only marginally higher due to the highly parallelized nature of modern GPUs, and it is compensated by the considerably fewer required iterations. Detailed discussion on the choice of the preconditioner can be found in Sec. 5.

##### Solving for multiple light sources in batches

On modern GPUs, the workload of solving for each light source individually (where the most expensive steps are a series of sparse matrix-vector multiplications) is usually small in comparison to their full computational capacity. In NIRFASTerFF, “batched” versions of both PCG and preconditioned BiCGStab solvers are implemented, where multiple light sources are solved simultaneously. This modification only requires two changes: (1) the sparse matrix-vector multiplications have now become sparse matrix-dense matrix multiplications, and (2) the vector update steps were performed simultaneously by distributing them among multiple (16 in the current implementation) CUDA streams.

By doing so, the GPU data throughput is increased, and the communication overhead of frequently launching small kernels is reduced; hence, the better efficiency of the solvers. This, of course, comes at the cost of increased memory use. To ensure that the algorithm fits into the GPU memory, the total required memory is first estimated, and if it exceeds the available amount, the looped version (i.e., solving for each light source in a loop) is used instead. If the looped version also fails, the CPU implementation will be called.

#### 2.2.2 Jacobian calculation

Jacobian matrix calculation is available for all mesh types (standard, fluorescence, and DCS) and is done using the adjoint method.^8^ It is worth noting that in NIRFASTerFF, Jacobians are always calculated in the voxel space, i.e., fluence data are first interpolated onto a regular voxelized grid before spatial integration (see Sec. 2.3.3).

Calculating the Jacobian in the voxel space can be computationally much more efficient because once both the direct field and the adjoint field are represented in the voxel space, it is reduced to a simple element-wise multiplication,^4^ thus avoiding the expensive integrations in the FEM space. Doing so also allows the user to easily choose, or even change, the resolution of the reconstruction basis. For example, one may use a low-resolution grid for a quick preliminary assessment before switching to a high-resolution grid for a more accurate final result.

The structure of the Jacobian matrix for standard problems is presented in a previous work,^10^ with the exception that the derivatives with respect to κ are now taken with respect to $\mu_{s}^{\prime }$ instead to better represent the problem.

In the fluorescence mesh, the full Jacobian matrix in the frequency domain is structured in a similar way. Suppose there are m source-detector pairs (i.e., channels) and p voxels in the new voxel space 
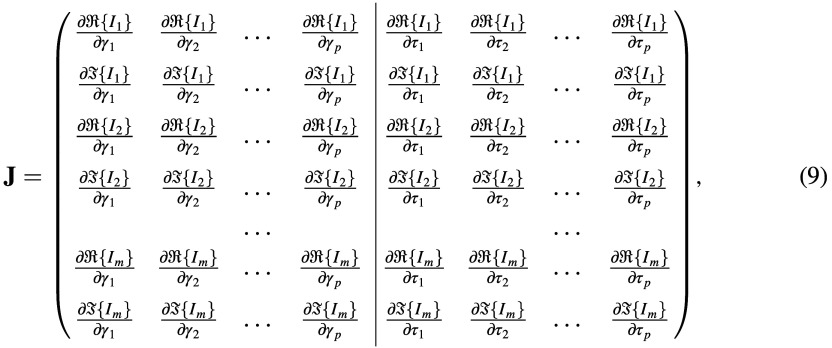
 where R and I are the real and imaginary operators, $I_{i}$ is the fluorescence re-emission intensity or re-emission intensity divided by excitation intensity (i.e., the “Born ratio”,^28^ the default), and $\gamma_{j}$ and $\tau_{j}$ are $\eta \mu_{af}$ and the fluorophore lifetime (in seconds) at voxel j. For CW data, the submatrix consisting of $\frac{\partial R\{I_{i}\}}{\partial \gamma_{j}}$ (i.e., rows 1,3,5,… of the left half) is returned because the data are by definition real and the derivative of its imaginary part is always zero.

On the DCS mesh, a time vector (the same as in the forward data calculation, see Sec. 2.3.3) must be provided, and a series of Jacobians are calculated at each point in the vector. Each one of them is structured similarly to the CW Jacobian on a standard mesh, i.e.,

$$J(\tau {)}_{ij}=\frac{\partial G_{1}(\tau {)}_{i}}{\partial (\alpha D_{b}{)}_{j}}\;\text{or}\;\frac{\partial \;\log(G_{1}(\tau {)}_{i})}{\partial (\alpha D_{b}{)}_{j}},$$ (10)

where the derivative of the logarithm is returned by default.

### 2.3 Organization

The NIRFASTerFF package is freely available (https://github.com/milabuob/nirfaster-FF) under the BSD license, with cross-platform support on Linux, macOS (both ARM and Intel chips), and Windows. On all platforms, the algorithms are accelerated using CPU multithreading via OpenMP. On Linux and Windows devices equipped with NVIDIA GPUs, CUDA acceleration is also available. The speed-critical algorithms (e.g., the FEM solvers) are implemented in C++ and CUDA and are provided as Python extension libraries, which are necessary to be downloaded in addition to the Python scripts. A compiled mesh generation program based on the CGAL library^29^^,^^30^ is also provided and is only needed by certain functions of the package. The use of the meshing program will be detailed in Sec. 2.3.2, and its source code is available in the folder mesher_src.

In this section, a high-level overview of the organization of the package is presented. More detailed documentation and examples are available on the GitHub repository: tutorials covering most of the commonly used applications can be found in the folder tutorials, in the Jupyter Notebook format. For advanced users, full API documentation can be found in the folder docs, in both HTML and PDF formats. Several example meshes can be found in the folder meshes. Note that one-based indexing and FORTRAN-ordered reshaping are used throughout the package for direct compatibility with the old NIRFAST(er) MATLAB toolbox.

#### 2.3.1 Modules and object-oriented design

The main package is contained in the nirfasterff folder, which consists of the following modules (detailed information can be found in the API documentation): base, forward, lib, meshing, inverse, io, math, utils, and visualize. For the user, the most important among them is the base. This is because NIRFASTerFF features an object-oriented design, where the most commonly used operations are implemented as methods of a “mesh” object and the results as associated “data” objects, which are classes of the base module.

**Fig. 4:**
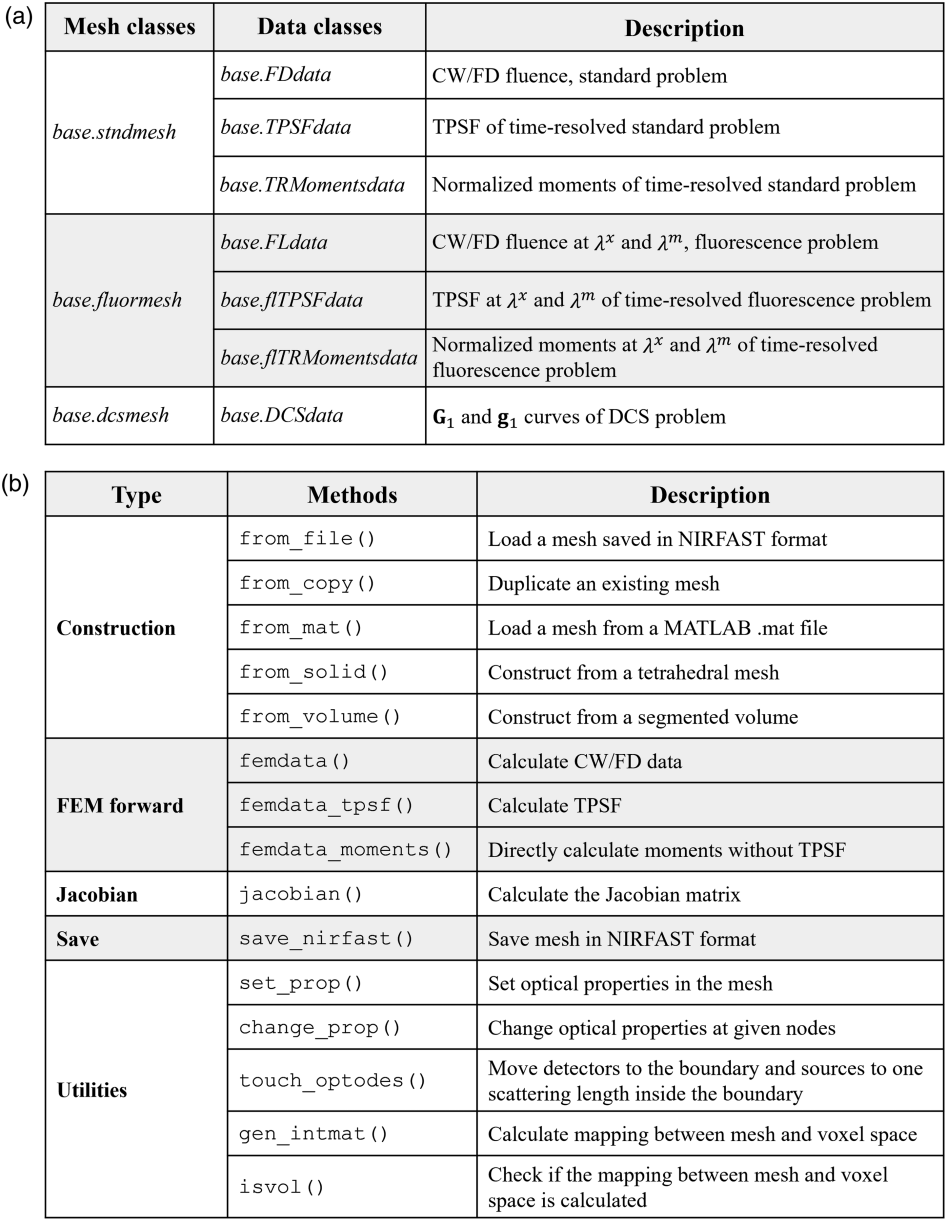
(a) Mesh classes and their associated data classes in nirfasterff.base module. In most applications, the operations will be centered around these classes. $\lambda^{x}$: excitation wavelength; $\lambda^{m}$: re-emission wavelength. (b) Summary of the methods of a standard mesh. Detailed explanations can be found in the API documentation.

The supported mesh types and their data types are summarized in Fig. 4(a). Using the standard mesh as an example, its attributes and available methods are illustrated in Fig. 4(b). The attributes and methods of the other mesh types are similar, and the details can be found in the API documentation.

#### 2.3.2 Input and output

Due to the object-oriented design of the package, it is always necessary to instantiate a mesh object of the appropriate type. As summarized in Fig. 4(b), there are five ways to set its attributes: (1) loading a mesh stored in the classic ASCII NIRFAST format; (2) loading a MATLAB.mat file that contains a NIRFAST mesh structure; (3) converting from a tetrahedral mesh stored in the TetGen^31^ format; (4) constructing from a segmented volume; and (5) deep copying a different mesh.

The meshes can also be saved in the classic ASCII NIRFAST format, which is fully compatible with the old NIRFAST(er) toolbox. This can be done using the method mesh.save_nirfast(). Detailed information can be found in the API documentation.

#### 2.3.3 FEM forward modeling and Jacobian calculation

For both standard and fluorescence mesh types, the package can calculate four types of forward data: CW, FD, TPSF, and statistical moments of time-resolved data. For the DCS mesh, only the electric field auto-correlation function ($G_{1}$ and $g_{1}$ curves) is calculated. This can be done by calling the appropriate method mesh.femdata*(). The available methods and their arguments are detailed in the tutorials and the API documentation. Those of the standard mesh are summarized in Fig. 4(b).

It should be noted that in the current version of NIRFASTerFF, only point sources are supported. To satisfy the isotropic source assumption in the diffusion equation, for each light source, unless explicitly disallowed, they are by default first projected onto the mesh surface, and subsequently moved inside by one scattering length ($1/\mu_{s}^{\prime }$ for standard and DCS meshes and $1/\mu_{s}^{x\prime }$ for fluorescence meshes). Similarly, all detectors are assumed to be point measurements and projected onto the mesh surface, albeit not moved inside.

It is recommended that a set of uniform grids in the x-, y-, and z-directions (for 3D models) is provided before FEM data calculation, in which case the returned internal fluences, TPSFs, moments, and $G_{1}$ curves will be represented in a voxel space defined by the grids.

Jacobian matrices (structures detailed in Sec. 2.2.2) can be calculated for all three (standard, fluorescence, and DCS) mesh types by calling the method mesh.jacobian(). As Jacobian matrices are calculated in the voxel space, providing the abovementioned uniform grids is necessary before Jacobian matrix calculation.

#### 2.3.4 Analytical solutions

In addition to the core functionalities based on FEM, NIRFASTerFF also calculates forward data using analytical solutions established in the literature.^4^^,^^32^^–^^34^ In the current version, only analytical solutions to the standard diffusion equation and correlation diffusion equation in homogeneous, semi-infinite media are available, subject to three types of boundary conditions, namely zero boundary condition (ZBC), extrapolated boundary condition (EBC), and partial current boundary (PCB).^33^ The supported problems are listed below

• CW/FD data of the standard problem
• TPSF of the standard problem
• $G_{1}/g_{1}$ curve of DCS.

The exact equations used for these problems can be found in Appendix B.

Using the analytical solutions can be done by calling

nirfasterff.forward.semi_infinite_*(args)

where * can be, CW, FD, TR, and DCS. The required arguments and the supported types of boundary conditions can be found in the API documentation. When the medium can be approximated as a semi-infinite space, e.g., a turbid phantom or a large piece of muscle, analytical solutions can be used to cheaply estimate photon distribution without the need to first construct a mesh. They are also commonly used in estimating optical properties of samples, e.g., by matching the shapes of theoretical and experimental TPSFs. An example of using the analytical solutions in optical property estimation is available in tutorial ParameterRecovery-TimeResolved.

## 3 Results

### 3.1 Fluence Calculation

**Fig. 5:**
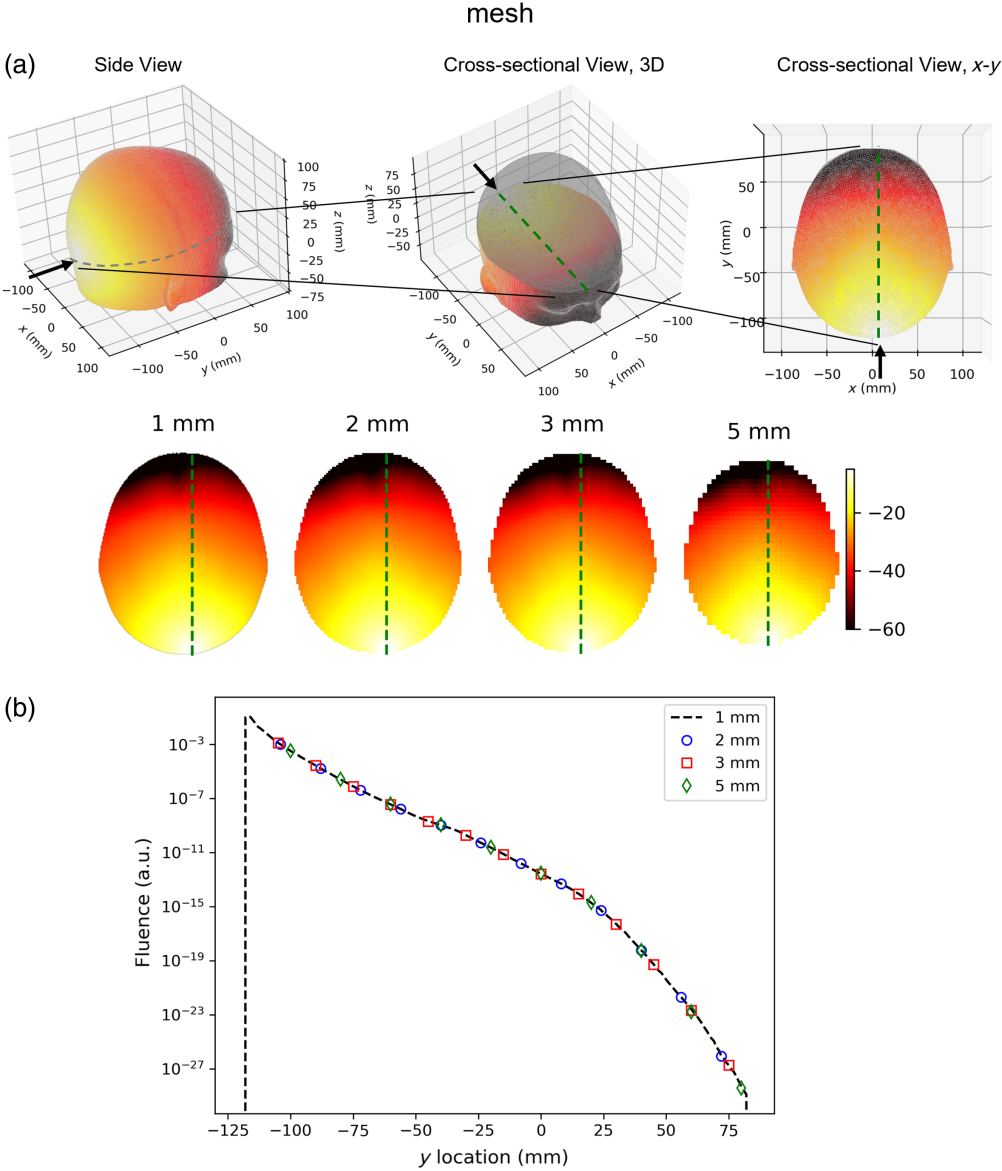
(a) Example result of the internal fluence in a human head mesh, horizontal section at the light source, log-scale. The same data were represented on the original mesh and grids of different resolutions. Black arrow: injection of photons; dashed green lines: lines along which data in panel (b) is taken. (b) Fluence along the y-axis cross-sectioned at the light source. The fluence results remain the same despite the different grid resolutions. Note that for clarity, only a subset of voxels is plotted.

A slice of an example result of the internal fluence in a human brain model is illustrated (in log-scale) in Fig. 5. Construction of the head model is detailed in the FullHead tutorial, available in the GitHub repository. In essence, the mesh was created from the segmented (skin, skull, cerebrospinal fluid, gray matter, and white matter) ICBM152 atlas, consisting of 688,557 nodes, 4,188,866 tetrahedrons, and 24 light sources placed over the visual cortex.^35^ The optical properties were chosen according to literature^35^ at 850 nm. The same data are represented on the original mesh as well as grids of four different resolutions (1×1×1  mm, 2×2×2  mm, 3×3×3  mm, and 5×5×5  mm). On a server with an NVIDIA Tesla V100 GPU (detailed in Sec. 4.1), the two steps (i.e., constructing the FEM matrices and solving the system) cost ∼3.2  s. The time needed to calculate the mapping from the mesh space to the voxel space is highly dependent on the number of tetrahedrons in the mesh and the size of the voxelized grid. For example, on a desktop PC with an Intel Core i7-13700K CPU, calculating the mapping from the above-mentioned head mesh to a 175×204×175 grid cost ∼15  s. However, this mapping only needs to be calculated once, and all the subsequent projections from mesh space to voxel space reduce to multiplications between a sparse matrix (i.e., the mapping matrix, number of voxels by number of nodes) and a dense matrix (i.e., data.phi, number of nodes by number of light sources), and typically costs less than a second.

As the mapping from the mesh space to the voxel space is done by linear interpolation, the internal fluence measured at a given location is, by definition, always the same regardless of the grid resolution, as shown in Fig. 5(b). However, if the total absorbed energy^36^ ($\int_{\Omega }\mu_{a}(r)\Phi (r)d\Omega$) is used as the metric, as shown in Table 1, quantization error does become nonnegligible when the voxel size is much larger than the size of the tetrahedrons, which is an expected artifact.

**Table 1:** Errors of total absorbed energy calculated with different grid resolutions, in comparison to its “ground truth” value calculated in the mesh space. Quantization error can be observed to increase with voxel size.

Grid size (mm)	1	2	3	5
Error (%)	0.95	0.42	5.6	11.6

### 3.2 Time-Resolved Data

#### 3.2.1 TPSF

**Fig. 6:**
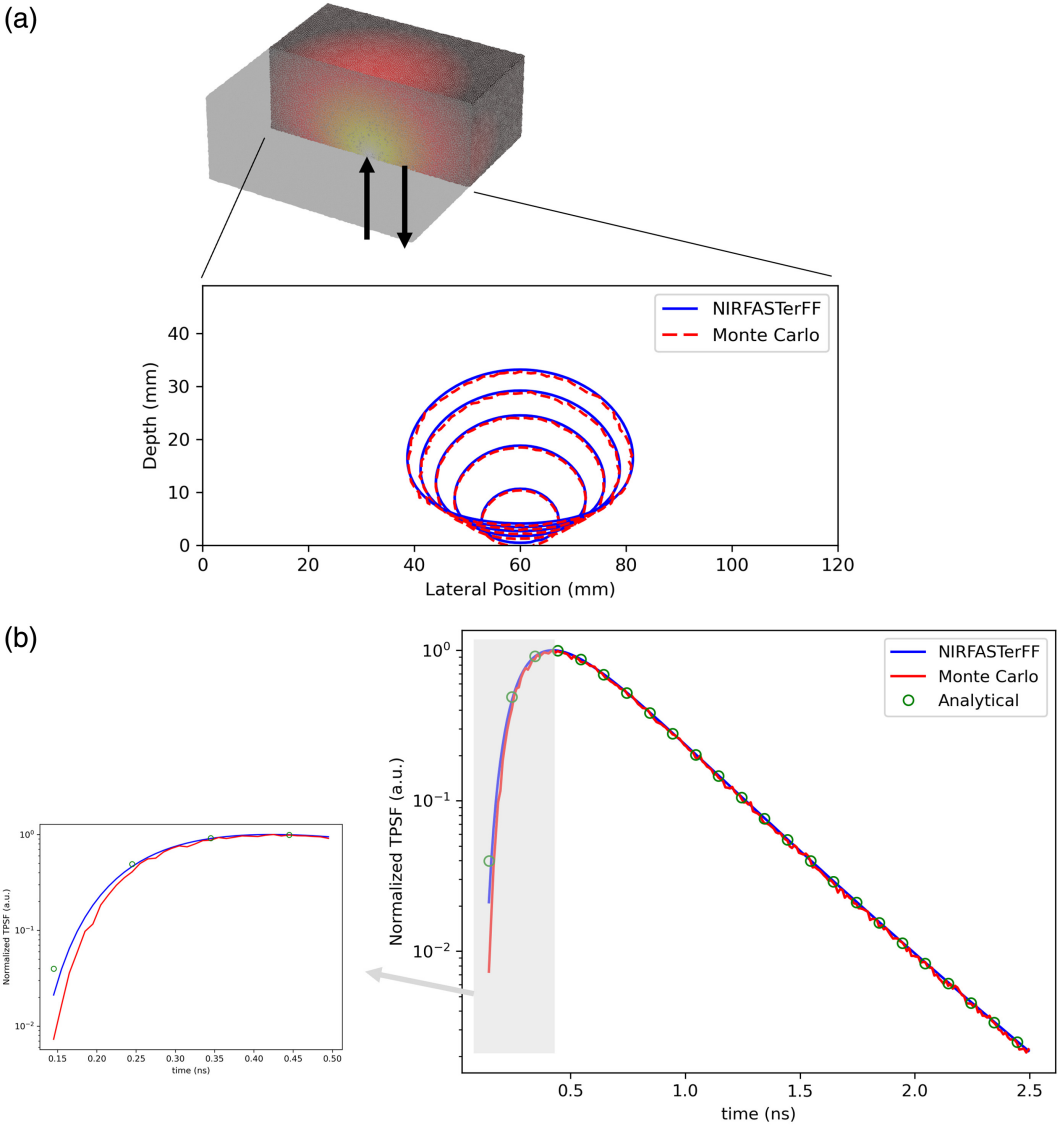
(a) TPSF result in a slab, compared with Monte Carlo simulation. The 3D figure shows the first moment as calculated using NIRFASTerFF and the cross-section at which the contours are calculated. Arrows indicate the locations of the light source and the detector. Contour lines illustrate the FWHM at 0.25,0.75,…,2.25  ns. (b) Comparison of boundary TPSF 2 cm from the light source: NIRFASTerFF, Monte Carlo, and analytical solution in semi-infinite media. The observed slight mismatches result from model inaccuracy (early photons) and statistical noise (late photons).

The FEM results were further validated against the Monte Carlo simulation in a thick slab model. In both FEM and Monte Carlo simulation, a homogeneous slab of 12×12×6  cm was used, with placed at the center of the bottom surface. The optical properties were assumed to be $\mu_{a}=0.01\;{\mathrm{mm}}^{-1}$, $\mu_{s}=10\;{\mathrm{mm}}^{-1}$, g=0.9, and n=1.37, where g is the anisotropy factor and n is the refractive index. The anisotropy factor was chosen to be representative of the human brain,^37^ and was incorporated into the Monte Carlo simulation via the Henyey–Greenstein phase function.^5^ In NIRFASTerFF, the reduced scattering coefficient, defined as $\mu_{s}^{\prime }=\mu_{s}(1-g)$, was used. The FEM mesh consists of 505,706 nodes and 3,010,644 tetrahedrons. TPSFs were calculated/simulated at 0.005 ns intervals up to 2.5 ns. For the Monte Carlo simulation, the GPU-accelerated pmcx package^5^ was used, and $10^{9}$ photons were launched. The number of launched photons was empirically and heuristically determined to have a good balance between simulation time and noise level. On the same V100 GPU, pmcx and NIRFASTerFF took ∼113.8 and ∼18.7  s, respectively. As shown in Fig. 6(a), comparisons of the full width at half-maximum (FWHM) contours at various time gates show good agreement between the FEM and Monte Carlo results, both spatially and temporally. Boundary measurements at 2 cm from the light source along the x-axis also show good agreement between the two methods and the analytical solution [see Fig. 6(b)]. Mismatches between FEM and Monte Carlo results can be observed at the early photons as the diffusion approximation is inaccurate for early time gates, and it is known to be difficult to accurately model the early photons using the FEM method.^38^ The Monte Carlo estimation of the late photons is slightly noisy because of the limited photons. Both results are again compared with the analytical solution in the semi-infinite medium, as calculated using NIRFASTerFF’s analytical solution function with an extrapolated boundary. The comparison of boundary data is shown in Fig. 6(b), and the mismatched results of the early photons are highlighted in the zoomed-in figure. The FEM solutions are slightly different from the analytical solution because of numerical errors (e.g., mesh density, step size, etc.), and the Monte Carlo results deviate far from both because the diffusion equation, on which both the analytical and the FEM solutions are based, is inherently inaccurate in this regime. The latter deviation is therefore expected and commonly observed in similar FEM–Monte Carlo comparisons.^5^ Another contributing factor is the different light sources used in the two methods: a pencil beam in Monte Carlo and an isotropic source (placed one scattering length below the surface) in NIRFASTerFF. In high-scattering media, e.g., the slab simulated here, the difference is only noticeable in the near field, but it can be more substantial when the scattering coefficient is low. Please see Sec. 5 for a detailed discussion. The errors of the first three normalized moments (see definition Sec. 2.1.4) relative to the analytical solution are 0.5%, 0.7%, and 1.0%. The errors relative to the Monte Carlo results are 2.0%, 0.64%, and 0.75%. The relatively large error in the zeroth moment estimation between NIRFASTerFF and Monte Carlo is likely caused by the mismatches observed for the early photons.

#### 3.2.2 Moments

**Fig. 7:**
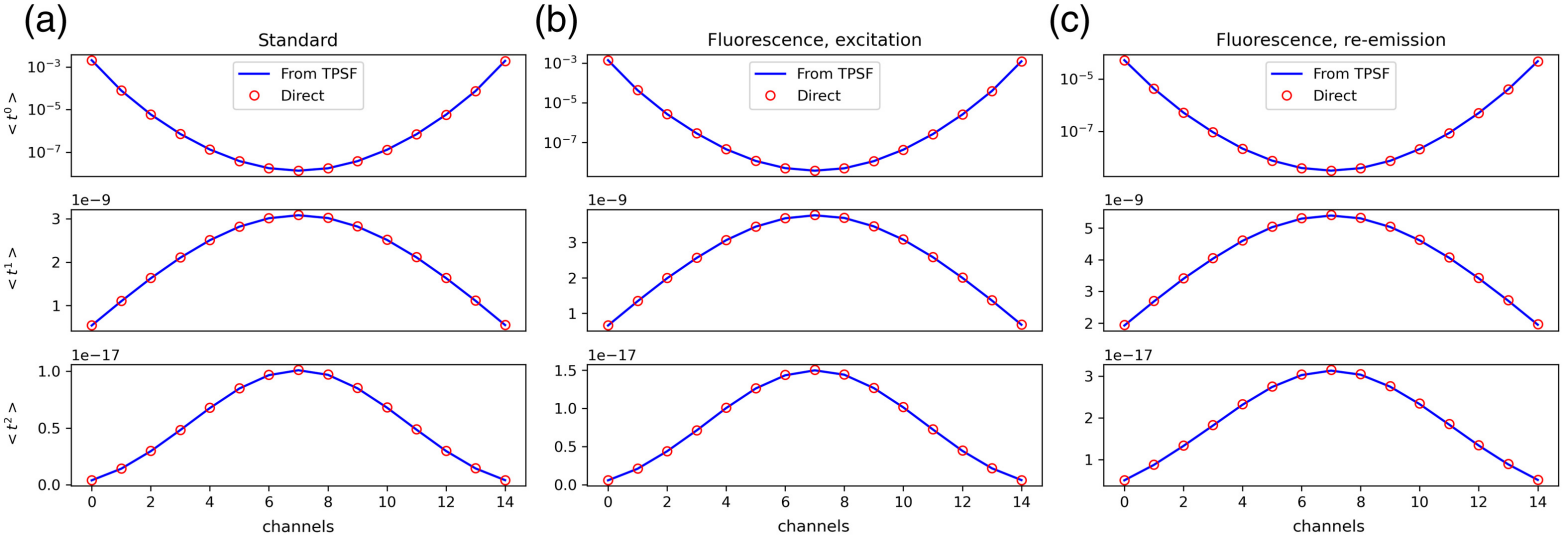
Comparison of normalized moments of time-resolved data, calculated from TPSF and Mellin transform. (a) Standard mesh. (b) Excitation data on a fluorescence mesh. (c) Fluorescence re-emission data on a fluorescence mesh.

The direct moment calculation was validated in both standard and fluorescence problems by comparing the results with those calculated from TPSF. To do so, the example homogeneous 2D disk meshes of 43 mm radius shipped with the package, circle2000_86_stnd and circle2000_86_fl, were used, with their optical properties specified in Table 2. Definitions of the variables can be found in Sec. 2.1. When calculating the TPSFs, the simulated time spans for the standard and fluorescence meshes were 10 and 15 ns, respectively, both with 10 ps resolution. As shown in Fig. 7 (shown only the first 15 channels, comprised of one light source and 15 equally spaced detectors along the circumference), normalized moments calculated using the two methods (integrated using TPSF and directly calculated using the Mellin transform) show excellent agreement in all the cases tested. Quantitatively, the maximum difference between the two methods was ∼2.9% due to numerical errors which can arise from, e.g., finite duration of simulation and finitely small step size.

**Table 2:** Optical properties used in the 2D disk meshes for validating the direct moment calculations.

		Standard mesh				
		$\mu_{a}$ (${\mathrm{mm}}^{-1}$)	$\mu_{s}^{\prime }$ (${\mathrm{mm}}^{-1}$)	n		
		0.01	1	1.33		

Fluorescence mesh						
$\mu_{a}^{x}$ (${\mathrm{mm}}^{-1}$)	$\mu_{s}^{x\prime }$ (${\mathrm{mm}}^{-1}$)	$\mu_{a}^{m}$ (${\mathrm{mm}}^{-1}$)	${\mu_{s}^{m}}^{\prime }$ (${\mathrm{mm}}^{-1}$)	γ (${\mathrm{mm}}^{-1}$)	τ (ns)	n
0.0089	1.3141	0.0062	1.2739	0.00018	1	1.33

### 3.3 DCS Data

**Fig. 8:**
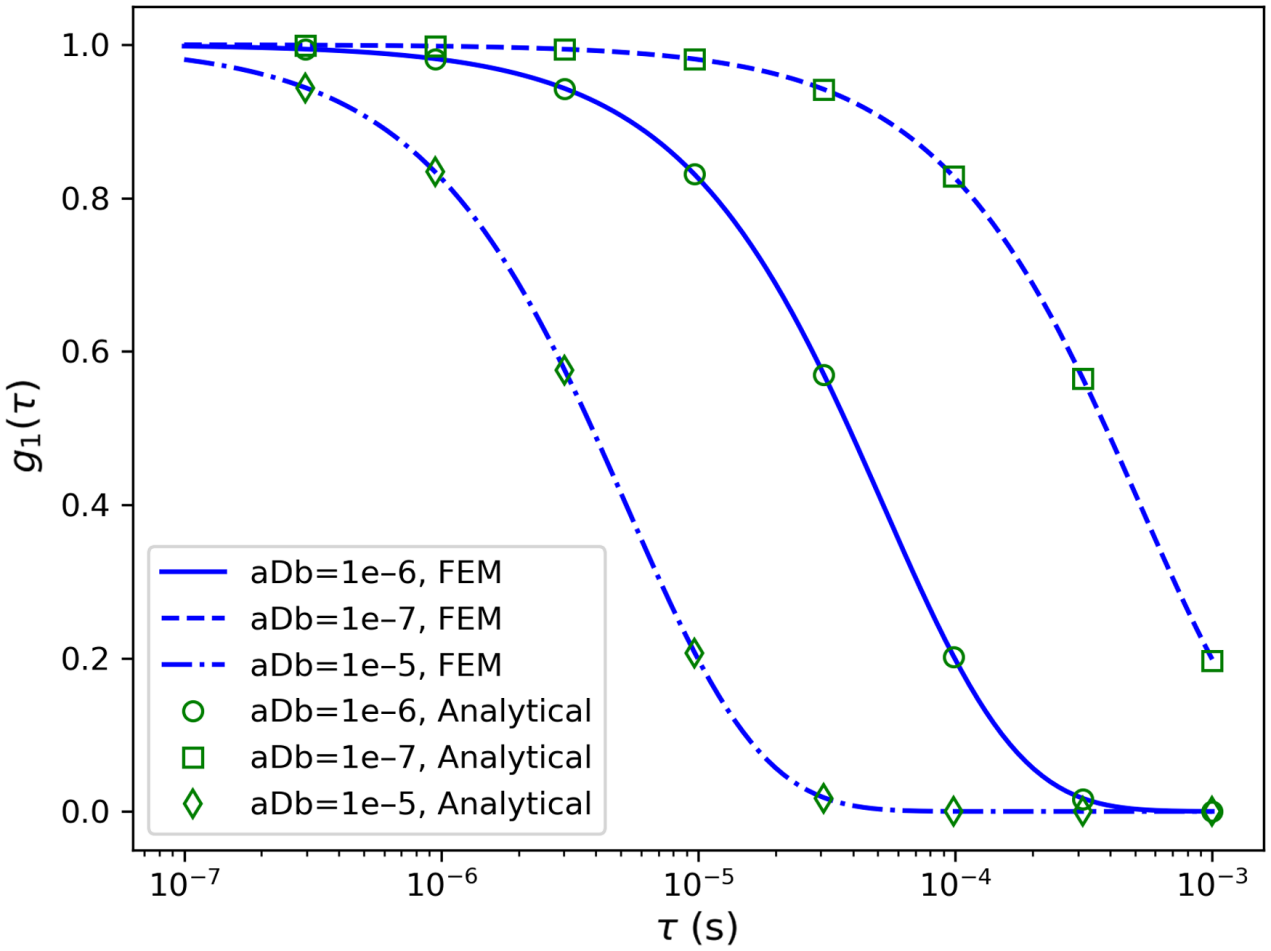
Comparison between $g_{1}(\tau )$ curves calculated using the FEM solver and the analytical solution in semi-infinite media.

To validate the calculation of the DCS data, the same slab model in Sec. 3.2.1 was used. The resulting $g_{1}(\tau )$ curves (2 cm away from the light source) were compared with the analytical solution in a semi-infinite medium with extrapolated boundary conditions, which was calculated using the analytical solution function of NIRFASTerFF. The wavelength was assumed to be 750 nm, and three different αDb values were used: $10^{-7}$, $10^{-6}$, and $10^{-5}\;{\mathrm{mm}}^{2}/s$. As shown in Fig. 8, the results from the two methods agree very well with each other. Quantitatively, the mean squared errors of the three curves were $4.7\times 10^{-4}$, $8.8\times 10^{-4}$, and $8.8\times 10^{-4}$, respectively.

### 3.4 Jacobian and Reconstruction

#### 3.4.1 Synthetic data

**Fig. 9:**
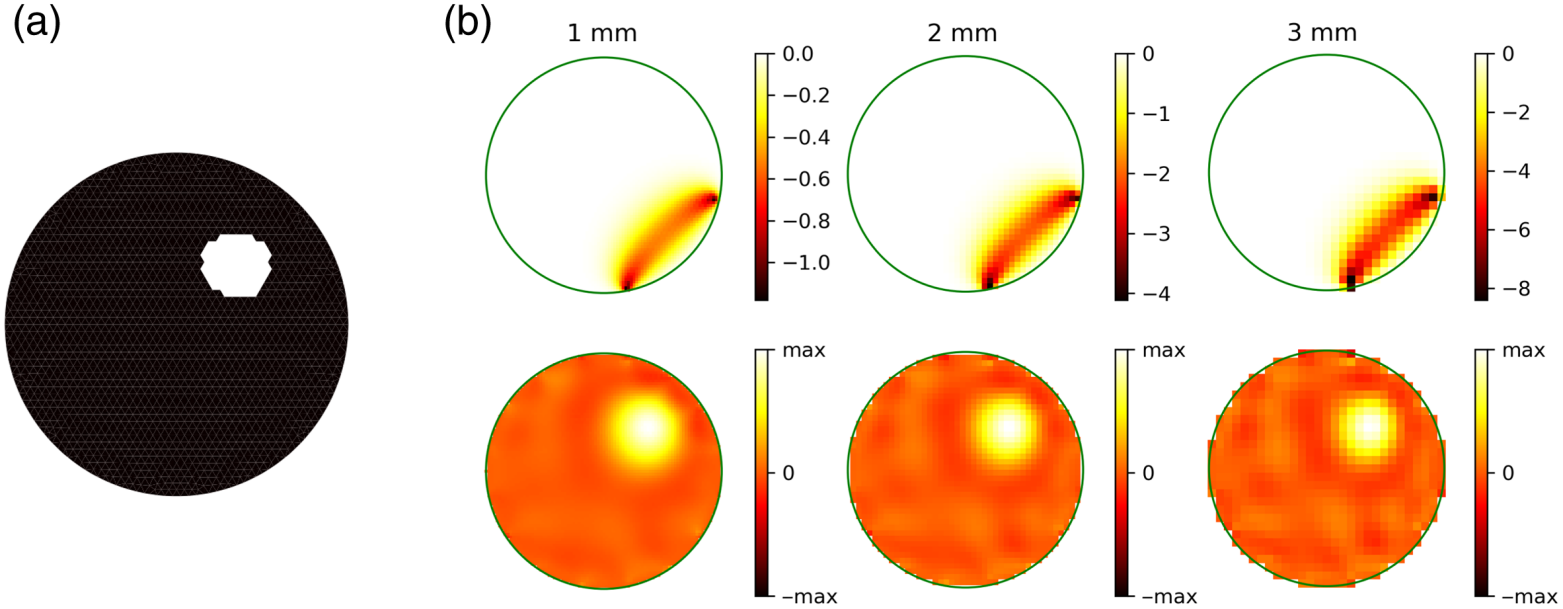
(a) Simple example: a 2D disk mesh with an anomaly of 5% $\mu_{a}$ increase. Plotted using the function nirfasterff.visualize.plotimage() (b) Effect of the grid resolution. Shown are the “banana” functions of an example channel at different grid resolutions and the corresponding reconstruction results. Green circles indicate the boundary of the mesh.

The effect of grid resolution on the resulting Jacobian matrices and reconstruction is illustrated using a simple example in Fig. 9. By definition (see Sec. 2.2.2), each row of the Jacobian matrix represents the sensitivity profile of a source-detector pair to each voxel in the space, and shown in the figure are the sensitivity profiles represented in the corresponding voxel spaces. Due to the characteristic curved shape of the sensitivity profiles, they are also commonly referred to as the “banana functions.”^3^^,^^4^ The same 2D mesh as mentioned above was used, and an anomaly of 5% $\mu_{a}$ increase was added. The log difference of the measured intensity resulting from the anomaly was corrupted with 5% white noise, before a Tikhonov regularization-based reconstruction was performed. This was done using the function nirfasterff.inverse.tikhonov(), with the regularizer chosen to be 10. This simplifying example shows the benefit of representing the Jacobian matrix in voxel space: one can easily change the preferred spatial resolution of the problem by changing the voxel size (e.g., high resolution for smoothness, or low resolution to reduce computational cost), and the activation pattern can be reconstructed regardless. Notice that when the voxel size increases, the values of the “banana” functions also increase accordingly. This is because the “banana” function indicates the sensitivity of a source-detector pair to each voxel, and when the voxel covers more volume of the space, the sensitivity also increases.

#### 3.4.2 Experimental data

DOT reconstruction was further tested on an experimental dataset (data “CCW1”, publicly available as part of the NeuroDOT package), which was collected during a human retinotopy experiment. In brief, the subject was given a visual stimulus of a rotating checkerboard wedge, and high-density DOT (24 sources and 28 detectors) was recorded over the visual cortex during the experiment at two wavelengths, 750 and 850 nm.^35^ The data were preprocessed following the suggested pipeline in the NeuroDOT package, which consists of conversion to optical density changes, linear detrending, bandpass filtering (0.02 to 1 Hz), superficial signal regression, and finally resampling to 1 Hz. Two voxelized ($2\times 2\times 2\;{\mathrm{mm}}^{3}$) Jacobian matrices, both defined over only the posterior part of the head where the optode grid is sensitive to, were calculated for the two wavelengths, respectively, with optical properties chosen according to the literature.^35^ When calculating the Jacobian matrices, the same head model in Sec. 3.1 was used. Performing reconstruction on only a chosen region of interest is another benefit enabled by voxelized Jacobian calculation, and is particularly useful in terms of reducing the problem size when the optode grid has limited coverage, as is the case in this example.

**Fig. 10:**
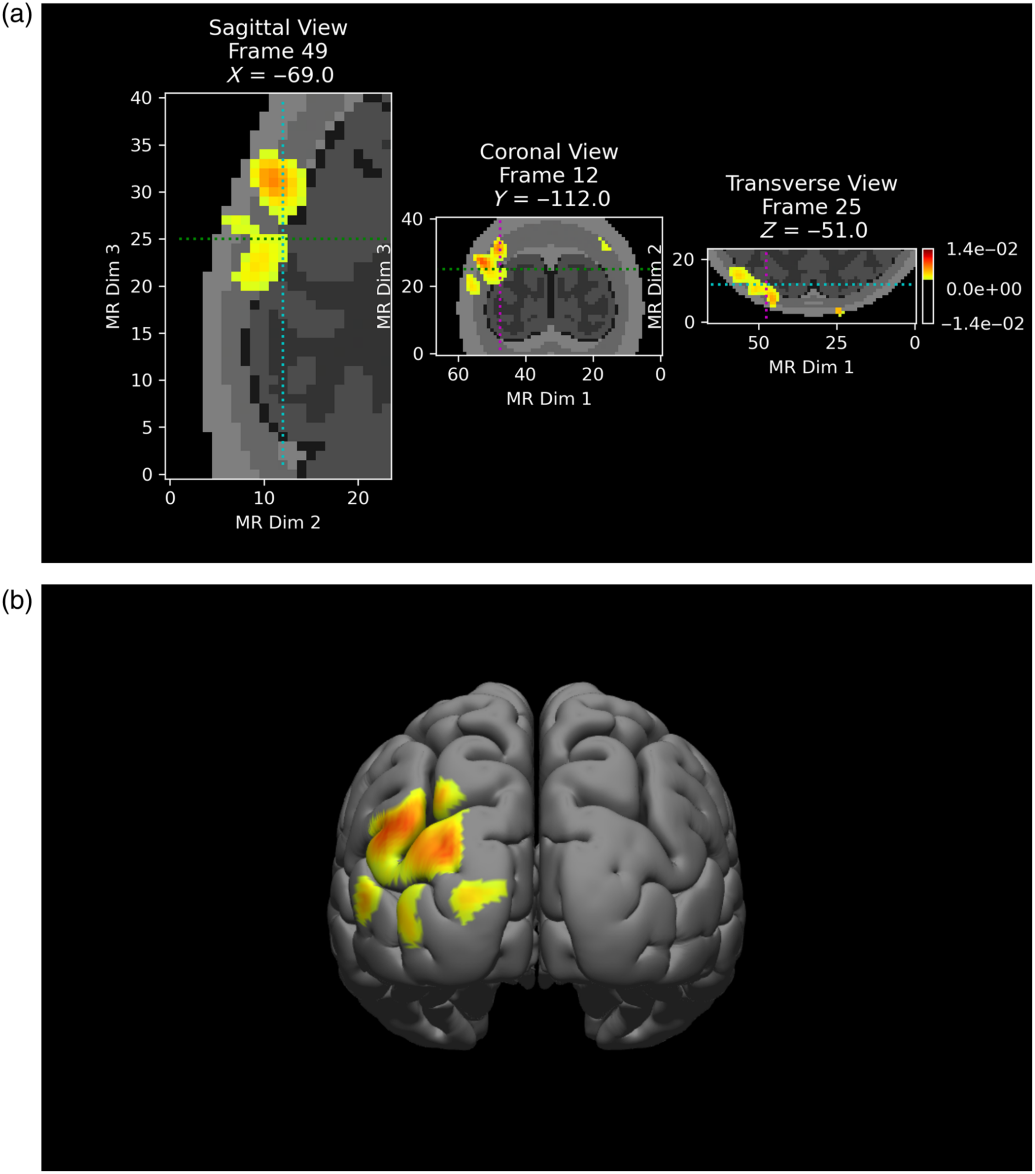
Reconstruction of ΔHbO, (a) represented as 3D slices in the voxel space, and (b) interpolated onto the cortical surface. Visualization was enabled by NeuroDOT and thresholded to show only the positive activities in both cases.

Utilizing the calculated Jacobian matrices, Tikhonov regularization^39^ was applied to the preprocessed data to reconstruct the absorption coefficient changes, during which process only source-detector pairs with a distance no greater than 40 mm and low noise (criteria detailed in NeuroDOT documentation) were used. Finally, the reconstructed absorption coefficient changes at the two wavelengths were converted to hemoglobin changes using Beer’s law and block-averaged. An example frame of the reconstructed oxygenated hemoglobin changes (ΔHbO) is shown in Fig. 10 in two representations: slices in the voxel space (co-registered with structural MRI) and interpolation onto the cortical surface. The full pipeline that was used to generate the results is available in the tutorial FullHead.

## 4 Benchmark

The performance improvement using the PCG and batched solvers was benchmarked against the BiCGStab solvers implemented in NIRFASTer through the same meshes used in a previous work,^7^ where the same MRI scan of a head was meshed into 13 different resolutions, ranging from 50,000 to 600,000 nodes. On each mesh, 158 light sources and 166 detectors were placed homogeneously across the whole head.

### 4.1 Improved Performance on GPU

Benchmarking of the CUDA solvers was performed on a server with a pair of Intel Xeon Bronze 3106 CPUs and an NVIDIA Tesla V100 GPU (32GB VRAM), running Ubuntu 22.04.3 LTS. MATLAB R2023b was used to run NIRFASTer, and Python 3.11.8 was used to run NIRFASTerFF. The same termination criteria of $10^{-12}$ relative error^7^ were used in all cases tested.

**Fig. 11:**
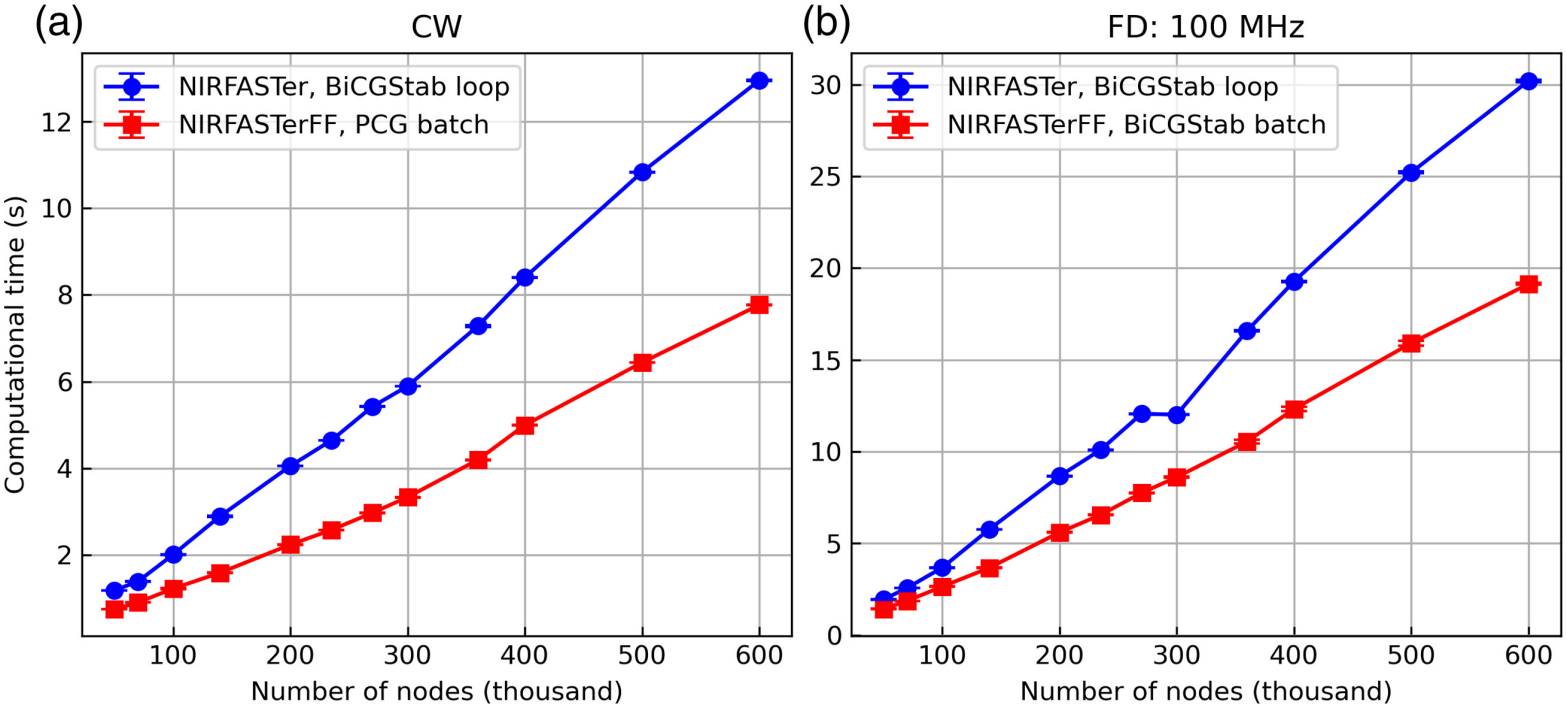
Comparison of the (a) CW and (b) FD (100 MHz modulation frequency) solvers on GPU, with $10^{-12}$ termination criteria. Solving for multiple light sources in batches significantly improves the solver efficiency, and the improvement is especially prominent when a faster PCG is used, as is shown in the CW example. Error bar: median absolute deviation.

For the CW solvers, in NIRFASTer, command data=femdata_stnd_FD(mesh,0) was benchmarked, and in NIRFASTerFF, command data=mesh.femdata(0)[0] was benchmarked. The two commands are functionally equivalent to each other, consisting of two steps: constructing the system matrices [Eq. (3)] and invoking the solver. It is therefore worth noting that the difference in performance results from both steps, although the solver is the main contributor, because the same algorithm was used for constructing the system matrices. These commands were chosen because the system matrix construction step is always necessary before the solvers are called, and as a result, they are more representative of a realistic use case. Both commands were repeated 10 times, and the median time consumption is shown in Fig. 11(a). The combination of using a more efficient PCG solver and maximizing parallelization by solving for multiple light sources in batches leads to a speed improvement of 35% to 45% among the meshes tested.

The same test was repeated for the FD data, where the modulation frequency was chosen to be 100 MHz, a commonly used frequency in commercial devices. The median time consumption is shown in Fig. 11(b). Although the same algorithm was used (despite the small implementation differences), the improved parallelization alone leads to a speedup of 25% to 37% among the meshes tested. A point of nonmonotonicity can be observed in the right panel likely because the mesh is poorly conditioned, which the NIRFASTer solver is known to handle poorly.^7^

**Fig. 12:**
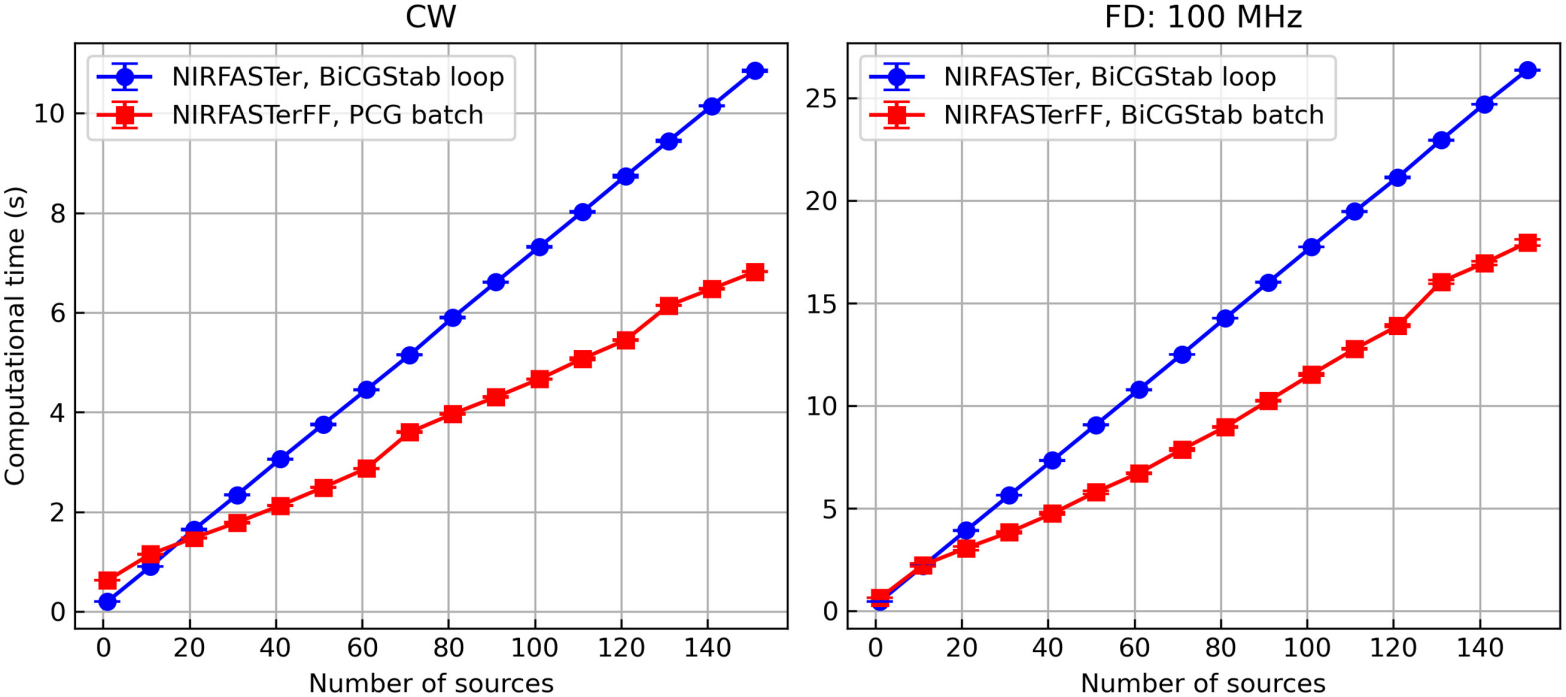
Computational time as a function of the number of light sources on GPU, with $10^{-12}$ termination criteria. The computational time of the NIRFASTerFF solvers scales more slowly than the old NIRFASTer solver and shows some nonlinearity due to the use of CUDA streams. Error bar: median absolute deviation.

The computational time as a function of the number of light sources was also quantified. The head mesh with 600,000 nodes was used, and in both NIRFASTer and NIRFASTerFF, the same subsets of 1,11,21,…,151 sources were used. The comparison was done in both CW and FD with a modulation frequency being 100 MHz. In this experiment, only the solvers (i.e., without the influence of system matrix construction) were timed to better illustrate the impact of solving for sources in batches. As shown in Fig. 12, the computational time of the looped BiCGStab solver as implemented in NIRFASTer, which solves for the light sources one by one, scales linearly with the number of light sources, as expected. The batched solvers scale much more slowly in comparison due to the improved parallelization (and also better algorithm efficiency in the case of PCG) and show some nonlinearity. The nonlinearity is likely caused by the use of CUDA streams, which causes a sharper increase in computational time whenever ⌊N_sources/N_streams⌋ (where N_streams=16 in the current implementation. See Sec. 2.2.1) increments. The benefit of the batched solver is not obvious when the number of light sources is low because of the lack of extra available parallelization and scheduling overhead.

### 4.2 Improved Performance on CPU

**Fig. 13:**
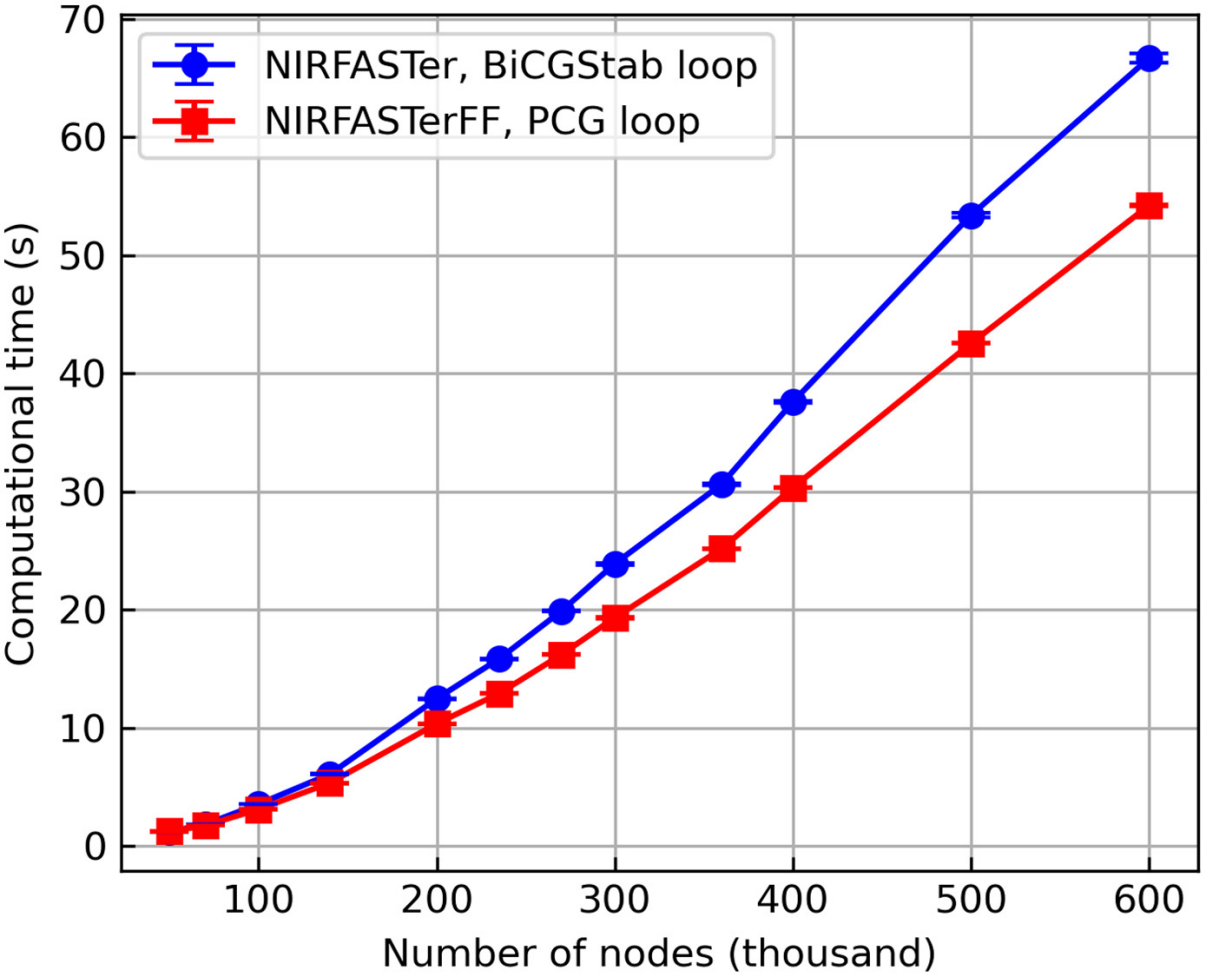
Comparison of the CW solvers on CPU, with $10^{-12}$ termination criteria. Both solvers loop over the light sources, but the new solver, which uses the more efficient PCG algorithm, has better performance, especially on larger meshes. Error bar: median absolute deviation.

The advantage of using PCG over BiCGStab was also benchmarked on the CPU. Analogous to the GPU benchmarking, commands data=femdata_stnd_FD(mesh,0) (in NIRFASTerFF) and data=mesh.femdata(0)[0] (in NIRFASTer) were used. This was performed on a desktop computer with an Intel Core i7-13700K CPU and 64 GB DDR5 RAM, running Ubuntu 22.04.5 LTS. MATLAB 2023a was used to run NIRFASTer, and Python 3.11.5 was used to run NIRFASTerFF. As shown in Fig. 13, although no extra parallelization (i.e., solving for multiple light sources in batches) was exploited, the use of the PCG algorithm alone leads to a considerable speed boost, especially on larger meshes. Quantitatively, among all the tested meshes, the largest improvement observed was ∼20%.

## 5 Discussion and Conclusion

This paper presented a new Python package for FEM-based photon modeling, NIRFASTerFF, applicable to the modeling of continuous-wave, frequency-domain, and time-resolved data in standard and fluorescence problems, as well as auto-correlation curves in DCS problems. The package is publicly available, easy to use, cross-platform, and very efficient, benchmarked to be up to 45% faster on GPU and 20% faster on CPU in comparison to the previous generation NIRFASTer toolbox. Support for more problems, e.g., photoacoustics and multiphysics modeling, will be added in future releases of NIRFASTerFF. Strictly speaking, the running time is also influenced by the interfacing language used (i.e., MATLAB versus Python). However, this influence is very small because the computational load implemented in MATLAB and Python is minimal, and the most computationally heavy parts are done by the C++ libraries.

Because NIRFASTerFF relies on solving the diffusion equation using FEM, it is only accurate when the medium has a much higher scattering coefficient than absorption coefficient, and the light source is, or can be well-approximated to be, isotropic. Although good agreement between Monte Carlo and FEM results was observed (see Fig. 6) despite the different light sources used (pencil beam versus isotropic), this is not in general true and can only be expected in high-scattering media, which is, fortunately, largely true for biological tissues.^37^ When anisotropy cannot be neglected, e.g., when simulating a directional light source in low-scattering media, or when there are sharp changes of refractive indices in the medium, NIRFASTerFF (as well as the other software based on similar principles) is no longer a suitable tool, and it is typically more appropriate to use Monte Carlo methods instead. It is indeed possible to model refractive index changes using FEM,^40^ but the implementation is nontrivial and remains to be further explored in future work. Another limitation results from the linear assumption made in the fluorescence problem formulation. As mentioned in Sec. 2.1.2, the formulation is only valid for weakly fluorescent systems, and the solution can become inaccurate even in biological systems when the approximation breaks down due to, e.g., high excitation intensity.

The performance of the PCG FEM matrix solver is strongly dependent on the design of the preconditioner, and it is generally difficult to theoretically find the optimal design. It is possible that the optimal choice of the preconditioner, as well as the other parameters of the CUDA kernel, needs to be tailored to the hardware used (known as “tuning”). This limitation can be potentially overcome using an automatic tuning mechanism,^41^ which searches for the best parameters on the hardware before invoking the main algorithm.

Similarly, the number of threads to use in OpenMP parallelization has a strong impact on the performance, and the optimal choice is also difficult to determine. This is especially true when the problem is large, in which case the memory bandwidth can be a bottleneck. It is possible that a different number should be chosen depending on the hardware and the problem (i.e., “tuning”). In NIRFASTerFF, the number of OpenMP threads is controlled by the function nirfasterff.utils.get_nthread(). This function is not exposed in the commonly used API, but the advanced user can tailor this function to their specific systems to maximize performance.

In the current release of NIRFASTerFF, GPU acceleration was implemented in CUDA and, therefore, supports only NVIDIA devices. It is possible to extend the GPU support to other vendors, e.g., AMD and Intel, using cross-platform frameworks such as OpenCL and SyCL. This remains to be investigated in future releases of NIRFASTerFF.

## 6 Appendix A: Derivation of Direction Moment Calculation in Time-Resolved Fluorescence Data

### 6.1 Moment Calculation

A typical scheme for solving the fluorescence diffusion equation is to first solve for the excitation field, and subsequently use it as the source term to solve for the re-emission field [see Eq. (4)]. The same FEM representation in Dehghani et al.^10^ is used, where $\left\{u_{1}(r),u_{2}(r),u_{3}(r),\ldots u_{n}(r)\right\}$ is the basis spanning space Ω.

Lemma 1. *Let* $f(r)=\sum_{i}a_{i}u_{i}(r)$ *and* $g(r)=\sum_{i}b_{i}u_{i}(r)$. *If* $\left\{u_{1}(r),u_{2}(r),u_{3}(r),\ldots u_{n}(r)\right\}$ *have limited support, and at least one of* f(r) *or* g(r) *changes slowly with* r *compared to the discretization of* Ω, *for every* i∈1,2,…,n, $\int_{\Omega }f(r)g(r)u_{i}(r)d\Omega \approx \underset{j}{\sum }a_{j}b_{j}\int_{\Omega }u_{i}(r)u_{j}(r)d\Omega$.

The k’th element of the new source vector in the FEM space is

$$q_{k}^{m}(\omega )=\int_{\Omega }\Phi^{x}(r,\omega )\frac{\eta (r)\mu_{af}(r)}{1+i\omega \tau (r)}u_{k}(r)d\Omega ,$$ (11)

$$=\int_{\Omega }(\underset{i}{\sum }\Phi_{i}^{x}(\omega )u_{i}(r))\frac{\gamma (r)}{1+i\omega \tau (r)}u_{k}(r)d\Omega ,$$ (12)

$$=\underset{i}{\sum }\Phi_{i}^{x}(\omega )\int_{\Omega }\frac{\gamma (r)}{1+i\omega \tau (r)}u_{i}(r)u_{k}(r)d\Omega ,$$ (13)

$$\approx \underset{i}{\sum }{\overset{˜}{\Phi }}_{i}^{x}(\omega )\int_{\Omega }u_{i}(r)u_{k}(r)d\Omega$$ (14)

where

$${\overset{˜}{\Phi }}_{i}^{x}(\omega )=\frac{\gamma_{i}\Phi_{i}^{x}(\omega )}{1+i\omega \tau_{i}},$$ (15)

and the last step results from the application of Lemma 1, which holds because $\Phi^{x}(r,\omega )$ changes slowly with r in comparison to dΩ in diffuse optics, when the FEM elements are sufficiently small. In this equation, $\Phi^{x}(\omega )=[\Phi_{1}^{x}(\omega ),\Phi_{2}^{x}(\omega ),\ldots {]}^{T}$ is the FEM solution to the excitation field, and $\gamma_{i}$ and $\tau_{i}$ are the values of γ(r) and τ(r) at the i’th node. It is important to note that this expression is valid only when the FEM basis is chosen to be $u_{i}(N_{j})=\delta_{ij}$, where $N_{j}$ is the position of the j’th node so that the FEM coefficients coincide with the nodal properties. If we further define matrix U with

$$U_{ik}=\int_{\Omega }u_{i}(r)u_{k}(r)d\Omega .$$ (16)

The new source vector is, therefore,

$$q^{m}(\omega )=U{\overset{˜}{\Phi }}^{x}(\omega ),$$ (17)

where the elements in ${\overset{˜}{\Phi }}^{x}$ are defined in Eq. (15).

The remaining derivation is adapted from the procedure for a standard system (i.e., no fluorescence) in Arridge and Schweiger.^23^ For a function f(t) with f(t)=0 for t<0, its Mellin transform is defined as

$$m_{n}=M[f(t);n+1]=\int_{0}^{\infty }t^{n}f(t)dt.$$ (18)

Calculation of the Mellin transforms for the excitation field, denoted as $m_{n}^{x}$, is the same as a standard system. Using Eq. (12) of Arridge and Schweiger,^23^ we have

$$\frac{\partial^{n}}{\partial \omega^{n}}\Phi^{x}(\omega ){|}_{\omega =0}=\frac{m_{n}^{x}}{i^{n}\sqrt{2\pi }}.$$ (19)

It can also be seen that, for element j,

$$\frac{\partial^{n}}{\partial \omega^{n}}\frac{1}{1+i\omega \tau_{j}}=(-1{)}^{n}\frac{(i\tau_{j}{)}^{n}n!}{(1+i\omega \tau_{j}{)}^{n+1}}.$$ (20)

Therefore, the n’th derivative of element j in ${\overset{˜}{\Phi }}^{x}(\omega )$ [as defined in Eq. (15)] is, using the general Leibniz rule

$$\frac{\partial^{n}}{\partial \omega^{n}}{\overset{˜}{\Phi }}_{j}^{x}(\omega ){|}_{\omega =0}=\gamma_{j}\overset{n}{\underset{k=0}{\sum }}(\begin{array}{c} n\\ k \end{array})(\frac{\partial^{k}}{\partial \omega^{k}}\Phi_{j}^{x}(\omega ))(\frac{\partial^{n-k}}{\partial \omega^{n-k}}\frac{1}{1+i\omega \tau_{j}}){|}_{\omega =0},$$ (21)

$$=\gamma_{j}\overset{n}{\underset{k=0}{\sum }}(\begin{array}{c} n\\ k \end{array})\frac{m_{k\{j\}}^{x}}{i^{k}\sqrt{2\pi }}(-1{)}^{n-k}(i\tau_{j}{)}^{n-k}(n-k)!,$$ (22)

$$=\gamma_{j}\overset{n}{\underset{k=0}{\sum }}\frac{n!}{k!(n-k)!}\frac{m_{k\{j\}}^{x}}{i^{k}\sqrt{2\pi }}(-1{)}^{n-k}(i\tau_{j}{)}^{n-k}(n-k)!,$$ (23)

$$=\frac{\gamma_{j}}{\sqrt{2\pi }}\overset{n}{\underset{k=0}{\sum }}\frac{n!}{k!}(-1{)}^{n-k}i^{n-2\;k}\tau_{j}^{n-k}m_{k\{j\}}^{x}.$$ (24)

Multiply both sides by $i^{n}\sqrt{2\pi }$, this becomes

$$i^{n}\sqrt{2\pi }\frac{\partial^{n}}{\partial \omega^{n}}{\overset{˜}{\Phi }}_{j}^{x}(\omega ){|}_{\omega =0}=\gamma_{j}\overset{n}{\underset{k=0}{\sum }}\frac{n!}{k!}\tau_{j}^{n-k}m_{k\{j\}}^{x}.$$ (25)

Consequently,

$$i^{n+1}\sqrt{2\pi }\frac{\partial^{n+1}}{\partial \omega^{n+1}}{\overset{˜}{\Phi }}_{j}^{x}(\omega ){|}_{\omega =0}=\gamma_{j}\overset{n+1}{\underset{k=0}{\sum }}\frac{(n+1)!}{k!}\tau_{j}^{n+1-k}m_{k\{j\}}^{x},$$ (26)

$$=\gamma_{j}m_{n+1\{j\}}^{x}+\gamma_{j}\overset{n}{\underset{k=0}{\sum }}\frac{n!(n+1)}{k!}\tau_{j}^{n-k}\tau_{j}m_{k\{j\}}^{x},$$ (27)

$$=\gamma_{j}m_{n+1\{j\}}^{x}+(n+1)\tau_{j}i^{n}\sqrt{2\pi }\frac{\partial^{n}}{\partial \omega^{n}}{\overset{˜}{\Phi }}_{j}^{x}(\omega ){|}_{\omega =0}.$$ (28)

Define

$$s_{n}=i^{n}\sqrt{2\pi }\frac{\partial^{n}}{\partial \omega^{n}}{\overset{˜}{\Phi }}_{j}^{x}(\omega ){|}_{\omega =0}.$$ (29)

The relationship in Eq. (28) can be represented in vector form as

$$s_{n+1}=\gamma ⊙m_{n+1}^{x}+(n+1)(\tau ⊙s_{n}),$$ (30)

where ⊙ denotes the Hadamard product. Now take the n’th derivative of the system equation with respect to ω for the fluorescence re-emission [that is, Eq. (3) with superscript m], and evaluated it at ω=0. Adapting Eqs. (16)–(18) of Arridge and Schweiger,^23^ this gives

$$A^{m}\frac{\partial^{n}\Phi^{m}(\omega )}{\partial \omega^{n}}{|}_{\omega =0}+inB^{m}\frac{\partial^{n-1}\Phi^{m}(\omega )}{\partial \omega^{n-1}}{|}_{\omega =0}=\frac{\partial^{n}q^{m}(\omega )}{\partial \omega^{n}}{|}_{\omega =0},$$ (31)

$$=\frac{\partial^{n}}{\partial \omega^{n}}U{\overset{˜}{\Phi }}^{x}(\omega ){|}_{\omega =0}.$$ (32)

If we denote the Mellin transforms of the re-emission field as $m_{n}^{m}$ and apply the relationship in Eq. (19), this becomes

$$A^{m}\frac{m_{n}^{m}}{i^{n}\sqrt{2\pi }}+in\;B^{m}\frac{m_{n-1}^{m}}{i^{n-1}\sqrt{2\pi }}=U\frac{s_{n}}{i^{n}\sqrt{2\pi }}.$$ (33)

This simplifies into

$$A^{m}m_{n}^{m}-nB^{m}m_{n-1}^{m}={\mathrm{Us}}_{n}.$$ (34)

When n=0, the scheme reduces to the CW solution to the coupled diffusion equation [Eq. (5)], which gives

$$\left\{ \begin{array}{l} m_{0}^{x}=(A^{x}{)}^{-1}q^{x}\\ s_{0}=\gamma ⊙m_{0}^{x}\\ m_{0}^{m}=(A^{m}{)}^{-1}{\mathrm{Us}}_{0} \end{array}.\right.$$ (35)

The higher-order Mellin transforms can subsequently be calculated by, for n=1,2,…

$$\left\{ \begin{array}{l} m_{n}^{x}=n(A^{x}{)}^{-1}B^{x}m_{n-1}^{x}\\ s_{n}=\gamma ⊙m_{n}^{x}+n(\tau ⊙s_{n-1})\\ m_{n}^{m}=(A^{m}{)}^{-1}(nB^{m}m_{n-1}^{m}+{\mathrm{Us}}_{n}) \end{array}.\right.$$ (36)

Finally, analogous to Arridge and Schweiger,^23^ the normalized moments (defined in Sec. 2.1.4) of fluorescence re-emission can be calculated by

$$\langle t^{n}\rangle^{m}=m_{n}^{m}⊘m_{0}^{m},$$ (37)

where ⊘ denotes element-wise division.

### 6.2 Proof of Lemma 1

$$\int_{\Omega }f(r)g(r)u_{i}(r)d\Omega ,$$ (38)

$$=\int_{\Omega }(\underset{j}{\sum }a_{j}u_{j}(r))(\underset{k}{\sum }b_{k}u_{k}(r))u_{i}(r)d\Omega ,$$ (39)

$$=\underset{j}{\sum }\underset{k}{\sum }a_{j}b_{k}\int_{\Omega }u_{i}(r)u_{j}(r)u_{k}(r)d\Omega .$$ (40)

Without loss of generality, assume that g(r) is the function that has slow variation. Because the FEM basis is chosen to have limited support, $a_{j}b_{k}\int_{\Omega }u_{i}(r)u_{j}(r)u_{k}(r)$ is only nonzero when the i’th, j’th, and k’th nodes are in the same finite element. When the finite elements are sufficiently small compared with the spatial variation of g(r), we have $b_{k}\approx b_{j}$. This leads to

$$\underset{j}{\sum }\underset{k}{\sum }a_{j}b_{k}\int_{\Omega }u_{i}(r)u_{j}(r)u_{k}(r)d\Omega ,$$ (41)

$$\approx \underset{j}{\sum }\underset{k}{\sum }a_{j}b_{j}\int_{\Omega }u_{i}(r)u_{j}(r)u_{k}(r)d\Omega ,$$ (42)

$$=\underset{j}{\sum }a_{j}b_{j}\int_{\Omega }u_{i}(r)u_{j}(r)(\underset{k}{\sum }u_{k}(r))d\Omega ,$$ (43)

$$=\underset{j}{\sum }a_{j}b_{j}\int_{\Omega }u_{i}(r)u_{j}(r)d\Omega ,$$ (44)

where we used the fact that in FEM, $\underset{k}{\sum }u_{k}(r)\equiv 1$, ∀  r by definition.

## 7 Appendix B: Analytical Solutions

All analytical solutions used in the NIRFASTerFF package are well-established results from literature, and they are presented in the section only for completeness and to serve the purpose as a reference. We use the same notation as in Sec. 2.1, except that the location vector r of the optical properties is no longer needed due to the homogeneity assumption. In the rest of the section, denote the radial distance to the light source as ρ, the depth into the semi-infinite space as z, and define $z_{0}=1/\mu_{a}+\mu_{s}^{\prime }$. In all the solutions presented in the subsequent discussion, it is necessary to first calculate β, the boundary reflection term defined in Eq. (2). In NIRFASTerFF, three different methods are provided

1. Exact Fresnel reflection^42^

Fresnel reflection coefficient for an incident angle θ is defined as

$$R_{F}(\theta )=\frac{1}{2}(\frac{n\;\cos\;\theta^{\prime }-n_{\text{out}}\;\cos\;\theta }{n\;\cos\;\theta^{\prime }+n_{\text{out}}\;\cos\;\theta }{)}^{2}+\frac{1}{2}(\frac{n\;\cos\;\theta -n_{\text{out}}\;\cos\;\theta^{\prime }}{n\;\cos\;\theta +n_{\text{out}}\;\cos\;\theta^{\prime }}{)}^{2},$$ (45)

where $\theta^{\prime }$ is the refractive angle given by Snell’s law. If define,

$$R_{\phi }=\int_{0}^{\pi /2}2\;\sin\;\theta \;\cos\;\theta R_{F}(\theta )d\theta ,$$ (46)

$$R_{j}=\int_{0}^{\pi /2}3\;\sin\;\theta \;\cos^{2}\;\theta R_{F}(\theta )d\theta ,$$ (47)

and

$$R_{\mathrm{eff}}=\frac{R_{\phi }+R_{j}}{2-R_{\phi }+R_{j}},$$ (48)

β can be calculated as

$$\beta =\frac{1+R_{\mathrm{eff}}}{1-R_{\mathrm{eff}}}.$$ (49)

2. Approximate Fresnel reflection^43^

If the Fresnel reflection coefficient is approximated using a two-step function, where

$$R_{F}(\theta )\approx \{\begin{array}{ll} \frac{(n-n_{\mathrm{out}}{)}^{2}}{(n+n_{\text{out}}{)}^{2}}≔R_{0}, & \cos\;\theta \geq \cos\;\theta_{c},\\ 1, & 0\leq \cos\;\theta <\cos\;\theta_{c}, \end{array}$$ (50)

(i.e., assuming $\theta \approx \theta^{\prime }$) and $\theta_{c}$ is the critical angle, the integrals can be simplified, and we reach

$$\beta =\frac{2/(1-R_{0})-1+|\cos\;\theta_{c}{|}^{3}}{1-|\cos\;\theta_{c}{|}^{2}}.$$ (51)

3. Groenhuis’s empirical approximation^34^^,^^44^

If the defined relative refractive index $n_{\mathrm{rel}}=n/n_{\text{out}}$, $R_{\mathrm{eff}}$ can be empirically approximated as

$$R_{\mathrm{eff}}=-1.440n_{\mathrm{rel}}^{-2}+0.710n_{\mathrm{rel}}^{-1}+0.668+0.0636n_{\mathrm{rel}},$$ (52)

and subsequently,

$$\beta =\frac{1+R_{\mathrm{eff}}}{1-R_{\mathrm{eff}}}.$$ (53)

### 7.1 Frequency Domain and Continuous Wave

Here, only the equations for the frequency domain solution are presented. The continuous wave solutions can be obtained by simply setting ω=0.

#### 7.1.1 ZBC and EBC

Define $k=\sqrt{\frac{\mu_{a}c-i\omega }{c\kappa }}$. The solution is given by^4^^,^^42^

$$\phi (\rho ,z)=\frac{1}{4\pi \kappa }(\frac{\exp(-kr_{1})}{r_{1}}-\frac{\exp(-kr_{b})}{r_{b}}),$$ (54)

where $r_{1}=\sqrt{(z-z_{0}{)}^{2}+\rho^{2}}$, and $r_{b}=\sqrt{(z+z_{0}+2z_{b}{)}^{2}+\rho^{2}}$.

The only difference between the ZBC solution and the EBC solution is the choice of $z_{b}$. Specifically, when ZBC is used, $z_{b}=0$, and when EBC is used, $z_{b}=2\beta \kappa$, where the calculation of β is presented above.

#### 7.1.2 PCB

Define $r_{2}=\sqrt{(z+z_{0}{)}^{2}+\rho^{2}}$ and let $z_{b}=2\beta \kappa$ (same as EBC), the solution is given by^42^

$$\phi (\rho ,z)=\frac{1}{4\pi \kappa }(\frac{\exp(-kr_{1})}{r_{1}}+\frac{\exp(-kr_{2})}{r_{2}}-\frac{2}{z_{b}}\int_{0}^{\infty }dl\;\exp(-l/z_{b})\frac{\exp(-k\sqrt{(z+z_{b}+l{)}^{2}+\rho^{2}})}{\sqrt{(z+z_{b}+l{)}^{2}+\rho^{2}}}).$$ (55)

### 7.2 DCS

The same solutions as the continuous wave problem are used, except that $\mu_{a}$ is replaced by $\mu_{a}+2\alpha D_{b}\mu_{s^{\prime }}k_{0}^{2}\tau$. This is done by matching the terms in Eqs. (1) and (5).

### 7.3 TPSF

Let the definitions of $z_{b}$, $r_{1}$, $r_{2}$, and $r_{b}$ follow from the frequency domain solution.

#### 7.3.1 ZBC and EBC

The internal fluence (z>0) is given by^33^

$$\phi (\rho ,z,t)=\frac{c\;\exp(-\mu_{a}ct)}{(4\pi \kappa ct{)}^{3/2}}[\exp(-\frac{r_{1}^{2}}{4\kappa ct})-\exp(-\frac{r_{b}^{2}}{4\kappa ct})].$$ (56)

The boundary reflectance (z=0) is given by^33^

$$R(\rho ,t)=\frac{1}{2}\frac{\exp(-\mu_{a}ct)}{(4\pi \kappa c{)}^{3/2}t^{5/2}}[z_{0}\;\exp(-\frac{z_{0}^{2}+\rho^{2}}{4\kappa ct})+(z_{0}+2z_{b})\exp(-\frac{(z_{0}+2z_{b}{)}^{2}+\rho^{2}}{4\kappa ct})].$$ (57)

Analogous to the frequency domain solution, the only difference between ZBC and EBC is the definition of $z_{b}$.

#### 7.3.2 PCB

The internal fluence (z>0) is given by^33^

$$\phi (\rho ,z,t)=\frac{c\;\exp(-\mu_{a}ct)}{(4\pi \kappa ct{)}^{3/2}}[\exp(-\frac{r_{1}^{2}}{4\kappa ct})+\exp(-\frac{r_{2}^{2}}{4\kappa ct})-\frac{2}{z_{b}}\int_{0}^{\infty }dl\;\exp(-l/z_{b})\exp(-\frac{(z+z_{0}+l{)}^{2}+\rho^{2}}{4\kappa ct})].$$ (58)

The boundary reflectance (z=0) is given by^32^

$$R(\rho ,t)=\frac{z_{0}\;\exp(-\mu_{a}ct)}{(4\pi \kappa c{)}^{3/2}t^{5/2}}\;\exp(-\frac{z_{0}^{2}+\rho^{2}}{4\kappa ct})T_{\mathrm{PCB}}(t),$$ (59)

where

$$T_{\mathrm{PCB}}(t)=\frac{1}{a}(1-\sqrt{\frac{\pi }{ab}}\;\exp(\frac{(1+a{)}^{2}}{ab})erfc(\frac{1+a}{\sqrt{ab}})),$$ (60)

$a=z_{0}\beta /ct$, b=4β/3, and

$$erfc(x)=\frac{2}{\sqrt{\pi }}(1-\int_{0}^{x}\;\exp(-\xi^{2})d\xi ).$$ (61)

## Data Availability

The NIRFASTerFF package is freely available under the BSD license at https://github.com/milabuob/nirfaster-FF. Codes for benchmarking and generating the figures can be requested from the authors.
